# Systems biology of bacterial nitrogen fixation: High-throughput technology and its integrative description with constraint-based modeling

**DOI:** 10.1186/1752-0509-5-120

**Published:** 2011-07-29

**Authors:** Osbaldo Resendis-Antonio, Magdalena Hernández, Emmanuel Salazar, Sandra Contreras, Gabriel Martínez Batallar, Yolanda Mora, Sergio Encarnación

**Affiliations:** 1 Programa de Genomica Funcional de Procariotes. Centro de Ciencias Genómicas-UNAM. Av. Universidad s/n, Col. Chamilpa, Cuernavaca Morelos, C.P. 62210. Mexico

## Abstract

**Background:**

Bacterial nitrogen fixation is the biological process by which atmospheric nitrogen is uptaken by bacteroids located in plant root nodules and converted into ammonium through the enzymatic activity of nitrogenase. In practice, this biological process serves as a natural form of fertilization and its optimization has significant implications in sustainable agricultural programs. Currently, the advent of high-throughput technology supplies with valuable data that contribute to understanding the metabolic activity during bacterial nitrogen fixation. This undertaking is not trivial, and the development of computational methods useful in accomplishing an integrative, descriptive and predictive framework is a crucial issue to decoding the principles that regulated the metabolic activity of this biological process.

**Results:**

In this work we present a systems biology description of the metabolic activity in bacterial nitrogen fixation. This was accomplished by an integrative analysis involving high-throughput data and constraint-based modeling to characterize the metabolic activity in *Rhizobium etli *bacteroids located at the root nodules of *Phaseolus vulgaris (*bean plant). Proteome and transcriptome technologies led us to identify 415 proteins and 689 up-regulated genes that orchestrate this biological process. Taking into account these data, we: 1) extended the metabolic reconstruction reported for *R. etli*; 2) simulated the metabolic activity during symbiotic nitrogen fixation; and 3) evaluated the *in silico *results in terms of bacteria phenotype. Notably, constraint-based modeling simulated nitrogen fixation activity in such a way that 76.83% of the enzymes and 69.48% of the genes were experimentally justified. Finally, to further assess the predictive scope of the computational model, gene deletion analysis was carried out on nine metabolic enzymes. Our model concluded that an altered metabolic activity on these enzymes induced different effects in nitrogen fixation, all of these in qualitative agreement with observations made in *R. etli *and other *Rhizobiaceas*.

**Conclusions:**

In this work we present a genome scale study of the metabolic activity in bacterial nitrogen fixation. This approach leads us to construct a computational model that serves as a guide for 1) integrating high-throughput data, 2) describing and predicting metabolic activity, and 3) designing experiments to explore the genotype-phenotype relationship in bacterial nitrogen fixation.

## Background

Biological nitrogen fixation carried out by *Rhizobiaceas *represents nearly 70 percent of the entire nitrogen transformation required for maintaining life in our biosphere. Simultaneously, nitrogen fixation driven by these bacteria constitutes an appealing and natural strategy for developing sustainable agricultural programs due to its cost-effectiveness in crop improvement and its more environmentally friendly effects in comparison to those produced by chemical fertilizers [1]. Based on these fundamental and practical issues, the study of bacterial nitrogen fixation is one active line of research that in the post-genomic era demands new paradigms capable of surveying in a systematic fashion the metabolic organization by which this process occurs in nature.

At a molecular level, symbiotic nitrogen fixation arises as a consequence of the coordinated action of a variety of genes, proteins and metabolites that in turn activate signal transduction cascades and transcriptional factors inside bacteroids. At the end of the day, the consequences are the activation and repression of certain metabolic pathways whose end products are required for counteracting the microenvironmental conditions prevailing inside nodules [2-4]. The advent of high-throughput technologies has fostered the genome scale analysis for bacterial nitrogen fixation, and the output data constitute valuable material in deciphering their metabolic organization at different biological layers [5,6]. Although some significant results have been achieved in interpreting the high-throughput data, their overwhelming numbers and heterogeneous composition represent a challenge for inferring biological knowledge in a coherent and systematic fashion. This challenge is, indeed, a central issue in systems biology, and its solution demands integrative efforts among genome scale data, physiological knowledge and computational modeling [7-11].

With the purpose of contributing to this integrative challenge, in this paper we present a systems biology description in bacterial nitrogen fixation. In particular, it integrates high-throughput technology and flux balance analysis in order to explore the metabolic activity of *Rhizobium etli *bacteroids while they fix nitrogen in symbiotic association with *Phaseolus vulgaris *(common bean plant) [11]. To survey the bacterial phenotype and sketch the genetic and metabolic profile during nitrogen fixation, transcriptome and proteome technologies were carried out for *R. etli *bacteroids selected at 18 days after inoculation with root plants of *P. vulgaris *(see details in experimental procedure and methods). We selected this interval of time based on experimental knowledge that has indicated it as an average for maximum enzymatic activity of nitrogenase in *R etli *bacteroids. To identify those genes with a significant role in nitrogen fixation, we accomplished a comparative analysis between the gene expression profile at the nitrogen fixation stage and under free-living conditions in *R etli*, this last condition mainly defined by succinate and ammonia as carbon and nitrogen sources, respectively (see methods). Simultaneously, the protein profile inside bacteroids was obtained, also at 18 days after plant inoculation. A set of genes with significant participation in bacterial nitrogen fixation was defined by combining those genes differentially expressed in the two physiological conditions--free life and nitrogen fixation-- and those codifying for the proteins detected inside bacteroids. This same set of genes served as our benchmark for extending the metabolic reconstruction for *R. etli *metabolism (*iOR*363) and evaluating the consistency of the metabolic capacities inferred by the *in silico *analysis [8]. To assess the predictive scope of the model, we qualitatively compared the metabolic activity predicted by constraint-based modeling against that which was deduced from the high-throughput data obtained for *R etli*. Overall, our study represents a significant effort toward the reconstruction of a systems biology platform for studying metabolic activity in bacterial nitrogen fixation. It is characterized by its capacity to integrate and describe high-throughput data and predict the metabolic mechanism underlying bacterial nitrogen fixation.

## Results

### High-throughput technology to guide the Metabolic Reconstruction

**Figure 1:**
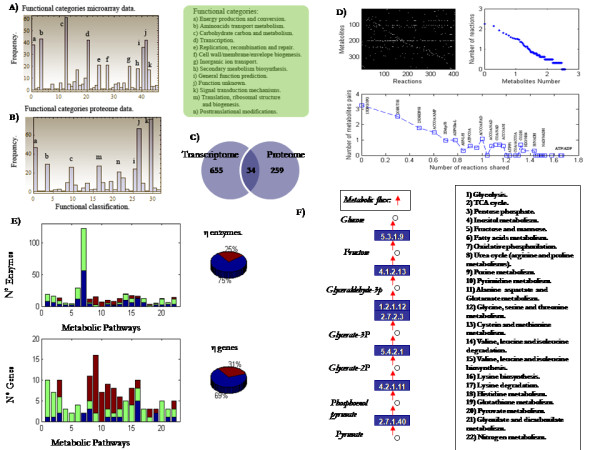
**Schematical view of data from high-throughput technology and constraint-based modeling**. (A) Functional distribution of up regulated genes in bacteroids. (B) Functional categories of proteome data. (C) Number of up regulated genes and proteins-coding genes identified by transcriptomics and proteomics. Overlapping region represents the number of genes that were identified by both technologies. (D) Topological properties of the metabolic reconstruction for *R.etli *(*iOR450)*. At the top from left to right: stoichiometric matrix and connectivity distribution (in log-log scale). At the bottom, metabolic pairs and its corresponding number of shared reactions (in log-log scale). (E) Figure in left side depicts the number of enzymes (genes) that were: 1) identified *in silico *but nor experimentally,(blue); 2) detected by both experimentally and *in silico *(green); and 3) experimentally detected but not observed *in silico *(red) along the 22 pathways listed in (F). Blue regions in right pies represent the overall percentage of genes and enzymes that simultaneously appear *in silico *and in high-throughput data. (F) A set of 22 metabolic pathways were used to assess the agreement between *in silico *and experimental results. Figure at left shows the activity of gluconeogenesis that emerged from the Flux Balance Analysis (FBA).

To characterize the gene expression during nitrogen fixation in *R.etli*, we compared each gene's activity in the free-living condition and in bacteroids driving nitrogen fixation selected at 18 days after inoculation with *P. vulgaris*. Data from microarray experiments were stored at the data depository *GEO *(http://www.ncbi.nlm.nih.gov/geo/) with access numbers GPL10081 for *R. etli *platform and GSE21638 for free life and symbiosis data. Even though a variety of sophisticated regulatory mechanisms may occur at diverse levels of biological organization [12], we have assumed that those genes with a significant over-expression indicate functional mechanisms for accomplishing nitrogen fixation. Under this criteria, we identified 689 genes (approximately 11% of the *R. etli *genome) whose transcriptional activity significantly increases during the biological process. To survey the role that these genes have in supporting nitrogen fixation, we classified them in accordance to the functional categories defined for *Rhizobiaceas *[13,14], see panel (A) in Figure 1 and Additional File 1. As expected, the majority of the *nif *and *fix *genes in bacteroids and other genes required for translation initiation, elongation, and termination were up-regulated inside nodules. Furthermore, our data suggest that the expression of genes forming part of translation initiation, elongation and termination machinery was not absent although it was significantly reduced in the nodule bacteria, a common observation reported in *Bradyrhizobium japonicum, Sinorhizobium meliloti *and *Mesorhizobium loti *bacteroids [15-17]. In accordance with the induction of cell-division inhibitor protein *minD*, a significant number of housekeeping genes down-regulate their expression at nitrogen fixation stages, and from microarray data we concluded that a slower rate of general metabolism, see Additional File 1.

To give a broader view of the biological activity inside the bacteroid, proteome analysis was conducted for *R. etli *bacteroids similarly recollected from nodules selected at 18 days after inoculation in root plants of *P. vulgaris *[18], see experimental procedure and methods. In total, proteome studies led us to identify and characterize 415 spot proteins in the bacteroids that suggested the expression of 293 genes during nitrogen fixation, see Figure 1 (B) and Additional File 2.

Both technologies--transcriptome and proteome--contributed to supply a broader biological landscape regarding bacterial nitrogen fixation. However, it is necessary to be aware of some differences in the experimental design underlying both technologies in order to integrate and interpret this data in a coherent fashion. While microarray technology resulted from a comparative analysis of two physiological conditions (free life and nitrogen fixation stages), proteome data identified the most abundant proteins present exclusively during nitrogen fixation stages. As Figure 1 (C) and Additional File 3 show, a scarce overlapping between the genes identified by both data sets is observed due to the experimental distinctness inherent in each technology. Thus, in order to identify those genes and enzymes with a relevant role in bacteroid metabolism and, in turn, form a set of genes that serve as a benchmark for computational assessment, we followed an integrative, rather than, selective strategy. Taking into account both sources of data, we hypothesized that up-regulated genes identified by microarray data and those genes that codify for the identified proteins potentially reveal those genes with a major role in nitrogen fixation. Under this assumption, both technologies led us to integrate a total of 948 genes that have a role in supporting bacterial nitrogen fixation, see Figure 1 and Additional File 1 and 2.

Functional classification of this set of genes ranged from enzymes participating in central metabolism and amino acid production to those maintaining specific pathways of nitrogen fixation such as glycogen and poly-β-hydroxybutyrate (PHB) biosynthesis. In addition, we identified enzymes participating in catabolism and anabolism of amino acids, chemotaxis, ribosome composition, RNA polymerase, DNA replication, nucleotide repairs, secretion systems and fatty acids metabolism. Moreover, a significant number of proteins participating as transporters reflects the intense metabolic crosstalk between plant and bacteroid; for instance, proteins participating in transport of small molecules, such as carbon, hydrogen, phosphate and sugar, fall under this classification, see panels (A) and (B) in Figure 1. We also identified proteins participating in the regulatory mechanism in nitrogen fixation, two components systems, transport and cell surface structure, energy transfer, cellular protection, and the transport and synthesis of polysaccharides. An extended discussion of the functional analysis that emerged from both technologies and its implication at a metabolic level can be reviewed in the Additional File 4.

### Expanding *Rhizobium etli *metabolic reconstruction and selecting pathways for its experimental assessment

The data generated by high-throughput technology constitutes a cornerstone in moving toward a descriptive analysis of nitrogen fixation. Despite the fact that this *top-down *scheme represents a valuable contribution to monitoring cell activity at a genome scale, complementary descriptions are required to integrate these data and survey how genetic perturbations affect nitrogen fixation in a systematic and quantitative fashion (*bottom-up *scheme). Among these quantitative schemes, constraint-based modeling is an appropriate formalism for exploring the cellular metabolic activity and guiding experiments to improve cellular behavior in a rational, coherent and optimal fashion [7,8,19,20]. In order to construct a *bottom-up *scheme for bacterial nitrogen fixation, our strategy consisted of three steps: 1) metabolic reconstruction for *R. etli*; 2) *in silico *modeling of nitrogen fixation, and 3) a cyclic assessment of computational predictions and experimental results.

In terms of metabolic reconstruction, proteome and transcriptome data were used to elaborate on the previous report for *R. etli *[8], thereby making some metabolic improvements and including new metabolic pathways absent in the previous version. To visually identify these metabolic reactions, we proceeded to represent the set of genes identified by high-throughput data and those from *iOR363 *reconstruction into each metabolic pathways defined in KEGG database. A comparative analysis among each pathway led us to visualize and highlight their differences. Consistent with the previous metabolic reconstruction, certain reactions were identified in the experimental set of data, while others led us to postulate the activity of new metabolic pathways that were absent in the previous reconstruction [8]. Specifically, high-throughput data strongly indicated the biological activity of fatty acid metabolism, and we therefore included this pathway in the metabolic reconstruction, see supplementary material. Overall, a set of 405 reactions and 450 genes made up the new metabolic reconstruction for *R. etli *(*i0R450*) with which *in silico *simulations and analysis were carried out. Topological properties that emerged from the updated metabolic reconstruction are shown in Figure 1 (D).

To evaluate the concordance between the metabolic activity predicted *in silico *and that interpreted from high-throughput technology, we selected 22 *KEGG *metabolic pathways [21] that had the highest number of genes experimentally detected by high-throughput data see Figure 1 (F). According to the KEGG database, these 22 metabolic pathways contain 311 genes for *R. etli *of which 76.7% were included in the metabolic reconstruction *iOR450*. This set of genes and their corresponding enzymes constituted the central core for evaluating the coherence between *in silico *predictions and high-throughput data interpretations. Even though *in silico *assessment relies on the activity of 22 metabolic pathways, *in silico *analysis of nitrogen fixation took into account all the reactions included in the metabolic reconstruction. This latter procedure will be valuable especially for exploring and predicting the metabolic role that additional pathways have on nitrogen fixation.

### Constraint-based modeling: evaluating the descriptive and predictive capacities of the metabolic reconstruction

Constraint-based modeling is useful for predicting the metabolic phenotype in microorganisms surviving in specific environmental conditions and/or subject to genetic perturbations [7,22]. With the purpose of evaluating the phenotype capacities of the metabolic reconstruction, flux balance analysis (FBA) was carried out for *R. etli *by imposing physical and chemical constraints to each metabolic reaction and using an objective function that mimics symbiotic nitrogen fixation [8], see method section. As a result of this analysis, a set of enzymes and genes with a significant role in nitrogen fixation was identified *in silico *as those that underlie the metabolic fluxes obtained from FBA. To quantify the agreement of experimental and computational interpretations, we defined a consistency coefficient representing the fraction of genes (*η^Genes^*) or enzymes (*η^Enzymes^*) predicted active by FBA and detected by high-throughput technology, see methods section. This parameter ranges from 0 to 1, with 1 representing the highest and 0 the lowest consistency between the genes (or enzymes) detected from high-throughput technology and predicted *in silico*. To evaluate the numerical value of these parameters and estimate the coherence between modeling outputs and high-throughput data during nitrogen fixation, an early metabolic simulation on *iOR450 *was carried out using the objective function originally suggested in a previous work, *Z^Fix ^*[8], i.e.

where glycogen, lysine, poly-hydroxybutyrate, alanine, aspartate and ammonium are denoted as *glycogen*[c], *lys*[c], *phb*[c], *ala*[e], *asp*[e] and *nh4*[e], respectively. All these metabolites are required to support an effective symbiotic nitrogen fixation [8], and their spatial location is indicated by [c] and [e] for cytoplasm and external compound. As a result of this simulation, we obtained a consistency coefficient of *η^Genes ^*= 0.6835 for genes and *η^Enzymes ^*= 0.702 for enzymes. Notably, this numerical value implied that 68.35% of the genes and 70.2% of the enzymes predicted *in silico *were consistently identified by high-throughput technology. To evaluate the statistical significance of this correlation, a hypergeometric test was applied in each case. In terms of enzymes, the coefficient reflected that of 74 enzymes predicted *in silico*, 52 were identified by high-throughput data. Meanwhile, the gene consistency coefficient indicated that of 237 expressed genes, 162 were identified experimentally. In both cases we concluded that these correlations were statistically significant: *p-value *= 8.59 × 10^-35 ^and *p-value *= 4.9 × 10^-64 ^for genes and enzymes, respectively.

### Improving predictability capacity of constraint-based modeling

These results encouraged us to proceed with an analysis of the *in silico *metabolic phenotype during nitrogen fixation, yet some improvements are desirable for ensuring a model with coherent interpretations and accurate predictions. To raise the qualitative agreement between *top-down *(high-throughput data) and *bottom-up *(*in silico *modeling) schemes, we therefore explored the possibility of finding an expanded objective function whose *in silico *phenotype improves the protein consistency coefficient η. To avoid this procedure from becoming a simple computational artifact without a biological foundation, we limited the search to those metabolites whose significant role in the bacterial nitrogen fixation were subject to strong experimental evidence. Thus, guided by a review in the literature, two metabolites were included in the objective function: *L-valine *and *L-histidine *both with a biologically meaningful role in nitrogen fixation. Supporting this assumption, mutagenesis made on the biosynthesis of branched chain amino acids, such as *L-valine*, has been shown to be defective in the initiation of nodule formation on host legumes [23]. In addition, we found evidence that *L-histidine *is a central compound participating in the mechanisms for regulating nitrogen fixation [12], and we noted that its inclusion in the objective function increased the agreement with high-throughput data. We therefore constructed a new objective function to mimic metabolic activity during nitrogen fixation in bacteria, it now integrated by

where boldface letters indicate those metabolites that were added to the previous objective function. Taking into account this implementation and simulating the flux distribution through FBA as described above, we obtained the following results during nitrogen fixation: *η^Genes ^*= 0.6948 and *η^Enzymes ^*= 0.7683, see Figure 1(E). In terms of enzyme activity this numeric value indicates that of the 82 metabolic reactions predicted *in silico*, 63 of them were consistently justified by high-throughput data (*p-val = 3.05 ×10^-64^*). Meanwhile the gene consistency coefficient indicated that of 249 expressed genes, 173 were identified by high-throughput data (*p-value *= 4.9 ×10^-64^).

Given this improvement, a detailed comparison between computational predictions and high-throughput data of the 22 metabolic pathways defined in Figure 1 (E) led us to distinguish three possible cases: the presence of 1) genes (enzymes) that were predicted *in silico *but not detected experimentally, 2) genes (enzymes) that were consistently observed in both schemes, and 3) genes (enzymes) that were experimentally detected but not predicted *in silico*, see Figure 1 (E). As explained in the methods section, *η *is related to the fraction of genes (enzymes) that were consistently observed in both schemes and constitutes the backbone of our modeling assessment. However, the biological explanation for the discrepancies described above (in cases 1 and 3) requires feedback assessment between modeling and experiments. For instance, these discrepancies could be reflecting the presence of post-transcriptional and post-translational regulation during nitrogen fixation and the design of proper experiments will be fundamental to discarding or accepting this hypothesis.

## Discussion

**Figure 2:**
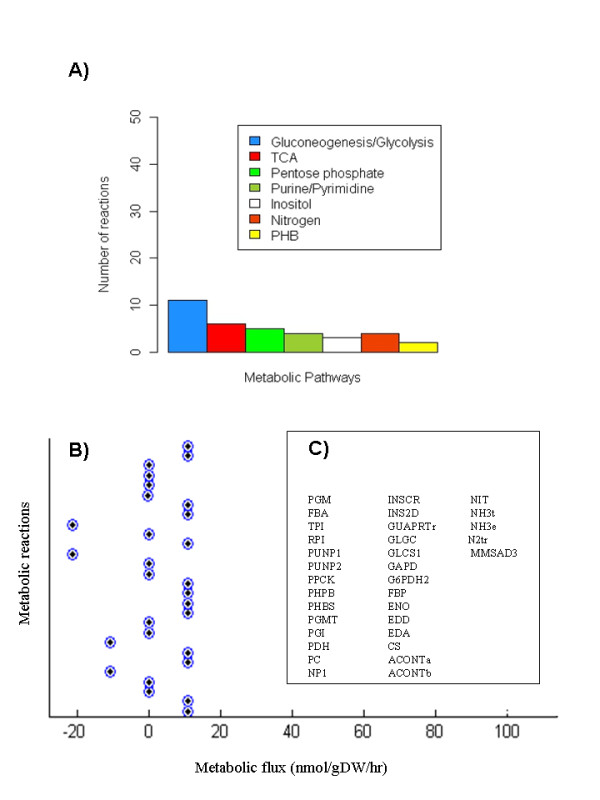
**Flux Variability Analysis (FVA)**. In panel (A) we depict the numerical participation of reactions with null variability along seven metabolic pathways included in the metabolic reconstruction. Reactions with null variability were defined as those whose upper and lower limit are equivalent. A fraction of reactions belonging to this classification are shown in (B). The set of reactions obtained by FVA are shown in (C). Here we have used the following abbreviations: PGM (phosphoglucomutase), FBA (fructose-bisphosphate aldolase), TPI (triose-phosphate isomerase), RPI (ribose-5-phosphate isomerase), PUNP1 (purine-nucleoside phosphorylase (Adenosine)), PUNP2 (purine-nucleoside phosphorylase (Deoxyadenosine)), PPCK(phosphoenolpyruvate carboxykinase), PHPB (acetoacetyl-CoA reductase), PHBS (PHB synthase), PGMT(phosphoglucomutase), PGI (glucose-6-phosphate isomerase), PDH (pyruvate dehydrogenase), PC (pyruvate carboxylase), NP1_r (nucleotide phosphatase), INSCR (inositol catabolic reactions (lumped)), INS2D (inositol 2-dehydrogenase), GUAPRTr (guanine phosphoribosyltransferase), GLGC (glucose-1-phosphate adenylyltransferase), GLCS1 (glycogen synthase (ADPGlc)), GAPD(glyceraldehyde-3-phosphate dehydrogenase), G6PDH2(glucose 6-phosphate dehydrogenase), FBP (fructose-bisphosphatase), ENO (enolase), EDD (6-phosphogluconate dehydratase), EDA (2-dehydro-3-deoxy-phosphogluconate aldolase), CS (citrate synthase), ACONTa (aconitase (half-reaction A, Citrate hydro-lyase)), ACONTb (aconitase (half-reaction B, Isocitrate hydro-lyase)), NIT (nitrogenase), NH3t (ammonia reversible transport), NH3e (Ammonium dissociation, extracellular), N2tr (Nitrogen exchange, diffusion) and MMSAD3 (methylmalonate-semialdehyde dehydrogenase (malonic semialdehyde)).

A coherent description between *in silico *modeling and high-throughput data is a primary goal for exploring the fundamental principles governing metabolism in *Rhizobiaceas *and predicting their phenotype behavior during nitrogen fixation. In this work we present a systems biology framework capable of exploring the metabolic activity of *R. etli *during nitrogen fixation in symbiosis with *P. vulgaris*. In particular, we present a genome scale model that integrates high-throughput data for describing, simulating and guiding experiments dealing with metabolic activity in bacterial nitrogen fixation. An important issue in constraint-based optimization analysis is the presence of alternate optimal fluxes, in other words the presence of a set of reactions--or flux distributions--that produce the same quantitative objective function. As a consequence of these alternate fluxes, the metabolic output of one pathway can be substituted by others such that macroscopic phenotype remains constant. Therefore, the distinction of the reactions with and without a range of variability is essential to guess the metabolic activity supporting biological phenotype. Hence, in order to characterize the core metabolic activity and compare our *in silico *metabolic interpretations with those emerged from high-throughput data, we carried out flux variability analysis (FVA) [24]. With the purpose to identify those reactions that represent the central core of metabolic activity along the set of alternate solutions, we limited our analysis to those reactions with a range of variability equivalent to zero. This set was such that the minimum and maximum flux variability for each reaction were equivalent and constituted our cornerstone for guiding the metabolic activity during the biological process. As depicted in Figure 2, the output of this analysis led us to identify some key reactions participating in some metabolic pathways required for sustaining bacterial nitrogen fixation. FVA was carried out with COBRA Toolbox [25]. As a consequence of this study, some concluding remarks immediately follow.

### Citric acid cycle

**Figure 3:**
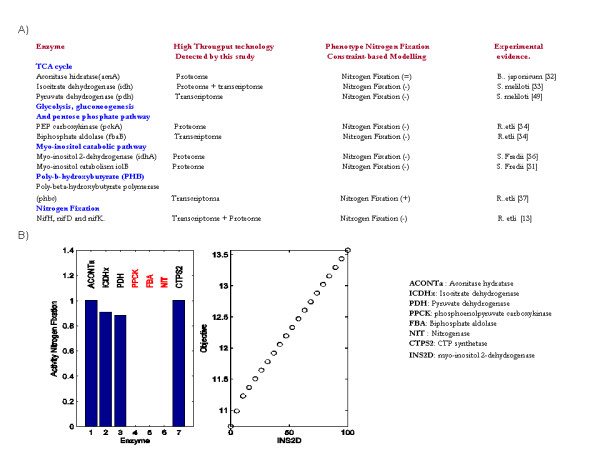
***In silico *assessment of gene knockout and phenotype variations on bacterial nitrogen fixation**. Panel (A) summarizes the benchmarks used to evaluate the *in silico *description of nitrogen fixation. Black and blue letter in first column indicates the silenced enzyme its corresponding metabolic pathway respectively. Second column indicates the technology by which the enzymes were identified in this study. Third column indicates the *Rhizobiacea *used to compare *in silico *prediction. Forth and fifth columns represent the computational phenotype and the reference supporting the computational result. Sign (+), ( = ) and (-) respectively denotes an increment, invariance and decrement in nitrogen fixation when mutation were accomplished. The in silico phenotype effect carried out by aconitase hydratase (ACONTa), isocitrate dehydrogenase (ICDHx), pyruvate dehydrogenase (PDH), phosphoenolpyruvate carboxykinase (PPCK), biphosphate aldolase (FBA), nitrogenase (NIT) and CTP-synthase (CTPS2) are summarized in left side of panel B. The robustness analysis accomplished for inositol catabolic reaction (INSCT) is shown in panel B.

Constraint-based modeling suggested that the TCA cycle is activated during nitrogen fixation by dicarboxylates which constitute the main carbon source in bacteroids [26], see Additional File 5 panel (B) in supplementary material. Consistent with this finding, eight proteins participating in the TCA cycle were detected in the *R. etli *bacteroid by proteome technology (FumC, FumB, LpdAch, SucB, SucA, SucC, Mdh and AcnA). To further assess this agreement, we applied gene deletion analysis to explore to what extend the deletion of some enzymes can qualitatively influence the activity of bacterial nitrogen fixation and if the predicted behavior is biologically coherent with knowledge reported in *Rhizobiaceas*, see method section. Thus, *in silico *gene deletion analysis accomplished on the metabolic reconstruction leads us to conclude that the aconitase hydratase (AcnA) mutant in *R. etli *is not lethal in nitrogen fixation. Despite the fact that this result has not been experimentally proven in *R. etli*, it has been validated in other *Rhizobiaceas *[27]. Furthermore, although isocitrate dehydrogenase (Icd) was not detected by high-throughput technology, *in silico icd *mutants in *R. etli *suggest a reduced phenotype on nitrogen fixation. This result is qualitatively in agreement with the fact that *icd *mutants on *S. meliloti *are symbiotically ineffective [28]. Similarly, constraint-based modeling concludes that a reduction of enzymatic activity in pyruvate dehydrogenase *(PDH) *induces a significant reduction in symbiotic nitrogen fixation but does not impair it as occurs in the case of *S. meliloti *bacteroids [29], see Figure 3 (B). This finding suggests that the role of PDH in the production of acetyl-coenzyme A can be replaced by alternative pathways in *R. etli *bacteroids [5]. The experimental assessment of this hypothesis for *R. etli *metabolisms is a central issue to explore in the future.

### Glycolysis, gluconeogenesis and pentose phosphate pathways

A common metabolic trait for some *Rhizobiaceas *is the intense activity of gluconeogenesis pathway [3]. In agreement with this finding, a significant number of gluconeogenic and glycolytic enzymes were identified by high-throughput technology, and constraint-based modeling consistently concluded that gluconeogenesis pathway was actively participating in nitrogen fixation.

Multiple isoforms of PEP carboxykinase (*pckA*) were detected by proteome technology, see Additional File 2, mirroring their pivotal role in nitrogen fixation and bacteroid differentiation. Thus, *R. etli *CE3 *pckA *mutant produces few nodules into which the infection threads do not appear to penetrate [30]. In qualitative agreement with this report, *in silico *mutation suggests that *pckA *is an essential gene for accomplishing nitrogen fixation in *R. etli*, see Figure 3.

In addition, 6-phosphogluconolactonase (*pgl*), glucose 6-phosphate dehydrogenas*e *(Zwf1), its chromosomal homolog (designated by zwf2) and one transaldolase (Tal) were detected by proteome, supplying evidence that pentose phosphate pathways can be actively participating in nitrogen fixation. Consistent with this finding, in fast-growing *Rhizobiaceas*, there is evidence that pentose phosphate and Entner-Doudoroff pathways work in coordinate action as the probable major routes for the metabolism of sugars [31].

As mentioned before, some glycolytic genes were identified by high-throughput data: two triosephosphate isomerases (TpiAch and TpiAf), one glyceraldehyde 3-phosphate dehydrogenase (Gap), one pyruvate kinase II (PykA), one 2-phosphoglycerate dehydratase (enolase), phosphoglycerate mutase *(pgm)*, and the bisphosphate aldolase (*fbaB*), see Additional File 1 and 2. Furthermore, there is experimental evidence that the genetic silence of *fbaB *in *R. etli *causes the development of sparse, empty nodules on root beans [30]. Consistent with this fact, computational gene deletion analysis carried out with this gene confirms that *fbaB *has a crucial role in supporting the metabolism of bacterial nitrogen fixation [30], see Figure 3(B). Even though these findings were not enough to postulate an active glycolytic cycle, they may suggest the metabolism of sugar intermediates via other pathways. For example, the presence of a specific transporter for glycerol-3-phosphate (*ugpAch1*, induced 3.88-fold by microarray analysis) indicates that this may be an important source for generating glycolytic intermediates. Similarly, the expression of 6-phosphogluconate dehydrogenase (Gnd) suggests the presence of an active pentose pathway, which is another potential channel for the metabolism of glycolytic intermediates.

### Myo-inositol catabolic pathway

Myo-inositol is one of the most abundant compounds in the soybean nodule, and accordingly, high-throughput technology successfully detected the presence of myo-inositol 2-dehydrogenase proteins (IdhA and IolB) encoding a *myo-inositol *protein in catabolism [32]. In agreement with this fact, computational analysis of the metabolism in *R. etli *suggests that a decrease of myo-inositol inside the nodule can reduce its capacity to fix nitrogen, see Figure 3(B). This result supports the hypothesis that the presence of myo-inositol in the nodule is essential for growth and maturation of the bacteroid and its metabolic inhibition can lead to both a nonfunctional bacteroid and the reduction of nitrogen-fixation activity [32].

### Poly-β-hydroxybutyrate and glycogen accumulation

While most of the bacteroid carbon supplied by the plant is channeled into energy production to fuel nitrogen reduction, in certain types of nodules some carbon is diverted by the bacteroids into the production of intracellular storage polymers composed of either glycogen or poly-β-hydroxybutyrate (PHB). Our simulations produced PHB, and consistent with our predictions, high-throughput analysis led us to identify the presence of three components related to its metabolic pathway: the polymerase PhbC (poly-beta hydroxybutyrate polymerase protein), a putative polyhydroxybutyrate depolymerase protein (detected by transcriptoma, see Additional File 1) and the acetyl-CoA acetyltransferase (beta-ketothiolase, *phbAch) *detected by proteome. Other reports confirm that metabolic fluxes in PHB and glycogen pathways are such that inhibition of one results in accumulation of the other, a property that was consistently observed by *in silico *modeling [8,33,34]. The precise role of PHB and glycogen during infection, nodulation, and nitrogen fixation and the factors that induce their accumulation are not yet determined. Future experiments dealing with these pathways are necessary to elucidate their role in bacterial nitrogen fixation.

### Nitrogen Fixation

To ensure the proper production of the ammonium required to establish an optimal bacterial-plant symbiosis, constraint-based modeling concludes that central genes involved in nitrogen fixation (*nif *and *fix *genes) are required for an optimal activity. Consistent with this fact, NifH, NifD and NifK were identified in proteome data and detected up-regulated in transcriptome analysis. In addition, an up-regulated gene expression was observed for *nifE *(nitrogenase reductase iron-molibdenum cofactor synthesis truncated protein), nifN (nitrogenase reductase iron-molibdenum cofactor synthesis protein), *nifX *(iron-molibdenum cofactor processing protein) and *nifB (FeMo *cofactor biosynthesis).

In *R. etli*, the *iscN *gene (Fe-S cofactor nitrogenase synthesis protein) is co-transcribed with *nifU *and *nifS*, and in conjunction, these genes were significantly up-regulated in bacteroids in comparison to bacteria under free-life condition (10.82, 3.92 and 1.99-fold, respectively). Furthermore, the *iscN *mutant in *R. etli *showed a significant reduction in nitrogen fixation [35]. Consistent with this report, *in silico *gene deletion analysis of those genes codifying for nitrogenase mostly reduces nitrogen fixation, see Figure 3.

### Amino acid metabolism and transport

A previous report suggests that *Rhizobiaceas *require the availability of 20 amino acids to establish an effective symbiosis with legumes [36]. Some amino acids are synthesized by *Rhizobiaceas *whereas the remaining are supplied by the host plant, a condition that appears to be plant-type specific. High-throughput analysis led us to identify certain proteins required for the synthesis of arginine, tyrosine, tryptophan, phenylalanine and lysine, the latter participating in the objective function defined in constraint-based modeling. On the other side, from the ABC-transporter proteins founded in nodule bacteria, thirteen were involved in amino acid transport, it strongly suggests that the uptake of amino acid is of particular importance in nitrogen fixation. The general amino acid ABC-transporter protein for AapJ (substrate binding protein) was detected by proteome analysis: the *aapJ *gene is part of the *aapJQMP *operon that exists in many *Rhizobiaceas *and has been described in detail in *R. leguminosarum *[37]. BraC1 and braC2, of the branched-chain amino acid ABC transporter, were detected in bacteroid by proteome and transcriptome technologies *(2.85 *fold). In *R. leguminosarum braDEFG *is required for alanine, histidine, leucine and arginine uptake [38] (two of which form part of the objective function associated with the metabolism of nitrogen fixation in our *in silico *model). Alternately, in *R. leguminosarum, braC *mutants are effective in *alanine *uptake (but are lacking in the uptake of the other three amino acids) [38]. Phenotype behavior for *braC *mutants has not been studied in *R. etli*, but there is evidence that *braD *and *braH *mutants were found to be deficient in glutamine uptake and respiration but proficient in nodulation and nitrogen fixation [30].

### Nucleotides metabolism

Purine and pyrimidine pathways are important during the nodulation processes given that most purine or pyrimidine auxotrophs in *Rhizobiaceas *are ineffective in symbiotic nitrogen fixation because they elicit pseudo-nodules devoid of infection threads [39]. Thus, for instance, the *purB *and *purH *gened in *Mesorhizobiumi loti *are involved in infection thread formation and nodule development in *Lotus japonicus *[40]. In addition, *purB *and *purH *mutants exhibited purine auxotrophy and nodulation deficiency in *L. japonicus *[40]. As Figure 2(A) and Additional File 5 panel (C) shows in the supplementary material, constraint-based modeling concludes that some enzymes in purine and pyrimidine pathways are actively participating in reaching an optimal symbiotic nitrogen fixation. Supporting this finding, several key enzymes were identified in bacteroids by proteome technology. Among them, we identified: phosphoribosylamine-glycine ligase protein (PurD), adenylosuccinate lyase protein (PurB), phosphoribosylformylglycinamidine synthetase protein (PurL), adenylosuccinate synthetase protein (PurA), IMP cyclohydrolase/phospho-ribosylaminoimidazole-carboxami-deformyltransferase protein (PurH), adenylate kinase (ATP-AMP transphosphorylase, Adk) and nucleoside-diphosphate-kinase protein (Ndk).

In the presence of adenine, only the *purH *mutant induced nodule formation, and the *purB *mutant produced few infection threads, suggesting that 5-aminoimidazole-4-carboxamide ribonucleotide biosynthesis catalyzed by PurB is required for the establishment of symbiosis. In addition, *purL *mutants in *S. fredii *HH103 strain does not grow in minimal medium unless the culture is supplemented with thiamin and adenine or an intermediate of purine biosynthesis [41]. Furthermore, gene expression of *purC1*, phosphoribosylaminoimidazole-succinocarboxamide (SAICAR) synthetase protein, *purUch *(formyltetrahydrofolate deformylase protein), *gmk2 *(*guanylate kinase *(GMP kinase protein) and *pyrE *(orotate phosphoribosyltransferase protein) were up-regulated inside bacteroids between 2.3 to 6.35 fold. In *S. meliloti*, nodule development in the case of *pyrE/pyrF *mutants did not reach the extent observed in the parental strain. These results suggest that some of the intermediates and/or enzymes of the *pyrimidine *biosynthetic pathway play a key role in bacteroid transformation and nodule development [42], information that should be taken into account for constructing an improved objective function and ensuring a proper computational description in future analysis.

### Fatty acids metabolisms

According to high-throughput data, metabolism of fatty acid can play a significant role in bacterial nitrogen fixation, this being in contrast to the drastic reduction of lipid biosynthesis observed in *B. japonicum *[43]. Thus, a variety of *fab *genes and proteins participating in fatty acid biosynthesis were detected by both methodologies (proteome and transcriptome). For instance, we detected by proteome the MccB subunit of methylcrotonyl-CoA carboxylase protein, acyl-CoA thiolase protein (FadA), enoyl-CoA hydratase protein (FadB1), enoyl-[acyl-carrier-protein] reductase (NADH) protein (FabI2) and S-malonyltransferase protein (FadD); and by transcriptome *fadB2 *was induced 3.09-fold. As these findings suggest, fatty acid metabolism could play an important role in bacteroid metabolism given that it can supply a variety of precursors such as components of the *rhizobial *membrane, lipopolysaccharides and coenzymes required in signal transduction. As opposed to the process in other *Rhizobiaceas *where fatty acids can be supplied by the host plant [43], we supply experimental evidence that bacteroids of *R. etli *synthesize and metabolize their fatty acids. The assessment of this hypothesis and the biological implications on bacterial nitrogen fixation constitute an avenue to experimentally verify in the future.

## Conclusions

In this study we present a systemic metabolic description of bacterial nitrogen fixation carried out by *R. etli *in symbiosis with *P. vulgaris*, at present the most complete study made in *Rhizobiaceas*. Collectively, high-throughput data suggest the following significant clues: 1) *R. etli *bacteroids are capable of synthesizing several amino acids through integrated carbon and nitrogen metabolisms. In addition, we observe the participation of some minor metabolic pathways such as myo-inositol catabolic pathway, degradation and synthesis of poly-b-hydroxybutyrate and glycogen. 2) Gene expression in bacteroids suggests the presence of a specialized transport system for sugars, proteins and ions. 3) An antioxidant defense mechanism based on peroxiredoxine, regulated by *nifA*, prevails during nitrogen fixation, as opposed to in free-living condition, where the mechanism is rooted in catalases [44]. 4) *R. etli *over-expresses genes and enzymes required in fatty acid and nucleic acid metabolism, contrary to other studies in bacteroids. Finally, 5) this study contributes a computational model that serves as a useful framework for integrating data, designing experiments and predicting the phenotype during bacterial nitrogen fixation, see Figure 3.

This systemic and integrative approach constitutes a valuable effort toward a systems biology description of the metabolism in bacterial nitrogen fixation; however, to increase our understanding and predictive accuracy some issues should be addressed in the future. Thus, particular attention should be directed toward those enzymes that were predicted metabolically active *in silico *but were not detected experimentally, and conversely, those enzymes that were detected experimentally but not *in silico*, see Figure 1(E). We expect that the study of these differences will be fundamental in postulating, verifying and uncovering mechanisms of regulation, while simultaneously confirming or improving hypotheses derived through *in silico *predictions.

Notably, even though the simulations have been carried out without a detailed numerical description of the coefficients *c_i _*in the objective function--see methods section--we have shown that the *in silico *model is capable of qualitatively predicting the activity of classic metabolic pathways and successfully describing some phenotype behavior in bacterial nitrogen fixation. Even though this represents a significant advance toward a systems biology description of bacterial nitrogen fixation, some improvements should be addressed in future. For instance, additional metabolites with a biological role in nitrogen fixation should be considered in order to obtain a more proper objective function that contributes to uncovering the role that less known metabolic pathways, such as nucleotides and fatty acid metabolisms, have on this biological process. As described here, these improvements will be guided by high-throughput data and the cyclic crosstalk between model and theory, a needed step in integrating, interpreting and generating biological hypotheses in a more accurate fashion.

Overall our study contributes to establishing the bases toward a systems biology platform capable of integrating high-throughput technology and computational simulation of bacterial nitrogen fixation. In particular, we envision that this metabolic reconstruction for *R. etli *(*iOR450*) will contribute to the rational design of optimal experiments that help us understand biological principles and identify those molecular mechanisms in order to improve this biological process, all this from a systems biology perspective.

## Methods

### Bacterial strains, growth conditions

The bacterial strain used was *R. etli *CFN42 wild type [11]. Culture media and growth conditions for *R. etli*, and plant experiments were accomplished as previously described in reference [45].

### Plant experiments

Three-day-old *Phaseolus vulgaris *cv. Negro Jamapa seedlings were inoculated with *R. etli *CFN42 strains as previously described by Peralta *et al*. [46]. After 18 days post-inoculation (dpi), nodules were picked out from the roots, immediately frozen in liquid nitrogen and stored at -70°C until further use. Bacteria were isolated from nodules and their identities verified by their antibiotic resistances.

### RNA isolation and microarray hybridization

Microarray experiments were carried out using three independently isolated RNA preparations from independent cultures and set of plants. Approximately 3 g of nodules were immersed in liquid nitrogen and macerated. Total RNA was isolated by acid hot-phenol extraction as described previously by de Vries *et al *[47]. For microaerobic free-living conditions, 50 ml of bacterial cell cultures were collected and total RNA isolated using a RNeasy Mini Kit (QIAGEN, Hilden, Germany). RNA concentration was determined by measuring the absorbance at 260 nm. The integrity of RNA was determined by running samples on a 1.3% agarose gel. 10 μg of RNA was differentially labeled with Cy3-dCTP and Cy5-dCTP using a CyScribe First-Strand cDNA labeling kit (Amersham Biosciences). Pairs of Cy3- and Cy5-labeled cDNA samples were mixed and hybridized to a Rhizobium_etli_CFN42_6051_v1.0 DNA microarray as described by Hegde et al. [48,49]. After washing, the arrays were scanned using a pixel size of 10 μm with a Scan Array Lite microarray scanner (Perkin-Elmer, Boston, MA). Three biological replicates with one dye swap were performed. We used real-time quantitative PCR to provide an independent analysis of gene expression for selected genes. Primer sequences and additional experimental protocols are reported in the supplementary material section.

### DNA microarray analysis

Spot detection, mean signals, mean local background intensities, image segmentation, and signal quantification were determined for the microarray images using the Array-Pro Analyzer 4.0 software (Media Cybernetics, L.P). Statistical treatment of microarray data was accomplished with bioconductors software (http://www.bioconductor.org/). Specifically, microarray normalization was carried out by applying the *maNormMain *function in the *marray *library. MA-plots before and after normalization are depicted in Additional File 5. Having normalized the gene expressions in the three experimental replicates, differentially expressed genes were identified by the following procedure. First, we calculate the average log-ration for each gene obtained from the three experimental replicates. Then, we obtained the standardized *z-score *of the log-ratio associated to each gene. The set of genes differentially expressed during nitrogen fixation was selected as those genes with a *z-score *higher than 1.65, see Additional File 5. The complete dataset used in the transcriptome analysis can be downloaded from GEO (http://www.ncbi.nlm.nih.gov/geo) with accession numbers: GPL10081 for *Rhizobium etli *platform and GSE21638 for free-life and symbiosis data.

### Verification by RT-PCR

We used real-time quantitative PCR to provide an independent assessment of gene expression for selected genes. The cDNA used for microarrays or freshly prepared cDNA was used as a template for Real-time PCR. Primer sequences used were as follows: *fabI2*-RECH000938f (5'-GTA TTG CCA AGG CCA TTC AT-3'), *fabI2*-RECH000938r (5'-CCC ACA GTT TTT CGA CGT TT-3') for the *fabI2 *gene. *idhA*-RECH003170f (5'-TTT CTT CAT GAC CCG CTA CA-3'), *idhA*-RECH003170r (5'-TTG ATC AGC TTG CCT TCC TT-3') for the *idhA *gene. *ppK*-RECH001491f (5'-TCC TGG CAC TGA ACA CTC TG-3'), *ppK*-RECH001491r (5'-GAG AAG GAA CTG GAC CAC CA- 3') for the *ppK *gene. *hisD*-RECH000581f 5'GAT CTG AAG CAA GCC ATT CC 3', *hisD*-RECH000581r (5'-ACA TAA TCG CCG ATG ACC TC-3') for the *hisD *gene. *nifH*-REPD00202f (5'-CCT CGG GCA GAA GAT CCT GA-3'), *nifH*-REPD00202r (5'-CAT CGC CGA GCA CGT CAT AG-3') for the *nifH *gene. *fixA*-REPD00224f (5'-ACA TCA ATG GGC GCG AGA TT-3'), *fixA*-REPD00224r (5'-TGT CGA TCT GCT CCG CCT TT-3') for the *fixA *gene. *cpxP2*-REPD00252f (5'-TCC GTG CCA TTT CAA AGA CC-3'), *cpxP2*-REPD00252f (5'-CCG CCA AAT GAG AAG ATT GC-3') for the *cpxP2 *gene. *hisC*-RE1SP0000233f (5'- CGA TGG CGA GAC AGC TAA AT-3'), *hisC*-RE1SP0000233r (5'-ATC ATC GCA ACG CTA TCT CC-3') for the *hisCd *gene. Each reaction contained 12.5 μl SYBR green PCR mastermix (Applied Biosystems), 3.5 μl H_2_O, forward and reverse primers in a volume of 5 μl, and template in a volume of 4 μl. PCR reactions were run with the ABI Prism 7700 sequence detection system (Applied Biosystems) using the following steps: 50°C for 2 min, 95°C for 10 min, followed by 40 cycles of 95°C for 15 s and 60°C for 1 min. The dissociation protocol was 95°C for 15 s, 60°C for 20 s, followed by ramp from 60°C to 95°C for 20 min. The transcript of the histidinol phosphate aminotransferase protein (*hisCd*) was used as an internal (unregulated) reference for relative quantification. This gene was selected as a reference because its expression is constitutive in all tested conditions (free live and symbiosis). Results of RT-PCR in real time were analyzed using the ΔΔCT method [49] and the data was presented like relative expression. All reactions were done by triplicate.

### Proteomics experiments

Bacteroids purification, protein extraction and two dimensional gel electrophoresis were done as previously described in [44]. Briefly, bacteroids were purified from root nodules by centrifugation through self-generated Percoll gradients. Bacteroid proteins were obtained by sonication at 24 kHz 1 min ON/1 min OFF for 5 cycles at 4°C in a Vibra Cell (Sonics, USA) in the presence of a protease inhibitor (Complete tablets, Roche Diagnostics GmbH, Mannheim, Germany). To further limit proteolysis, protein isolation was performed using phenol extraction. Two dimensional gel electrophoresis (2D-PAGE), was performed like previously described. Gels were stained with Coomasie Blue G-250, scanned with PDI image analysis system, and analyzed with PD-Quest software (Bio-Rad Laboratories, Inc, Hercules, CA.). Selected spots from preparative 2-D gels were excised, digested and the proteins were identified by PMF MALDI-TOF using a Bruker Daltonics Autoflex, following the same methodology mentioned in [44]. The experiments were performed three times. Selected spots from Coomassie stained preparative 2-D gels were excised and processing automatically using the Proteineer SP spot picker and DP digestion robots (Bruker Daltonics, Billerica MA). Mass spectra were obtained using a Bruker Daltonics Autoflex (Bruker Daltonics Bellerica, Mass. USA) operated in the delayed extraction and reflectron mode. Spectra was externally calibrated using a peptide calibration standard (Bruker Daltonics 206095). Peak lists of the tryptic peptide masses were generated using FlexAnalysis1.2vSD1Patch2 (Bruker Daltonics). The search engine MASCOT server 2.0 was used to compare fingerprints against Rhizobium etli CFN42, NC_007761.1, pA, NC_007762.1, pB, NC_007763.1, pC, NC_007764.1, pD, NC_004041.2, pE, NC_007765.1, pF, NC_007766.1 with the following parameters: one missed cleavage allowed, carbamidomethyl cysteine as the fixed modification and oxidation of methionine as the variable modification. We accepted those proteins with scores greater than 50 and a p < 0.05. Proteome data associated with this manuscript can be downloaded from http://ProteomeCommons.org Tranche using the following hash:

BY/eCcVjwTWN1+m+2ArvJ0QVnesGx5Ekgd4wUOASACfm/ueNl7YI3iLf4xz0lnGsepV5LkpMWOQOrZtjYExlNpQkIBcAAAAAAAABjA = =

### High-throughput technology and its use for extending metabolic reconstruction and simulating nitrogen fixation

With the purpose of establishing an integrative description between modeling and experimental data, we extended the metabolic reconstruction for *R. etli *by including those reactions whose enzyme activity were supported by high-throughput data. Thus, the fatty acids metabolism was included in the metabolic reconstruction, and some metabolic improvements were made along the network. Additional File 6 enlist the main abbreviations used along this paper. Additional File 7 in supplementary material contains a detailed description of the reactions included in this new metabolic version (*iOR450*). Overall, the updated metabolic reconstruction for *R.etli *consists of 377 metabolites and 450 genes codifying for enzymes participating in 405 metabolic reactions. The gene-protein reaction association for the entire metabolic reconstruction, lower and upper bounds and reversibility information associated to each reaction are shown in Additional File 7.

### Constraint-based modeling

Metabolic flux distribution supporting nitrogen fixation in *Rhizobium etli *was predicted *in silico *by constraint-based modeling [8]. Briefly, simulations were carried out assuming a steady-state condition for metabolic fluxes and by constructing a mathematical function that mimics nitrogen fixation. This objective function, *Z^Fix^*, consists of certain key compounds required for sustaining nitrogen fixation and others required for mimicking the physiological conditions prevailing in the boundaries of the nodules. Thus, objective function was mathematically written as a linear combination of these metabolites (*X_i_*) and their contribution to nitrogen fixation was weighted by coefficients (*c_i_*), which for simplicity's sake were all selected as a unit. With the purpose of obtaining a computational profile of metabolic fluxes, we assumed that the metabolic state of the bacteroid during nitrogen fixation is one that optimizes the objective function, *Z^Fix^*. This latter issue was solved by taking into account linear programming and considering that the fluxes are constrained by their enzymatic and thermodynamic capacities,

where *S_i,j _*represents the entries of the stoichiometric matrix, *v_j _*is the metabolic flux of the *j-th *reaction and *α_j _*and *β_j _*account for thermodynamic and enzymatic constraints, see Additional File 7. Linear programming was carried out using the *Tomlab *optimization package called from COBRA toolbox in *Matlab *[25].

### External metabolites considered for flux balance analysis

In order to explore the phenotype capacities of the bacteria metabolism, we included in the reconstruction certain exchange and sink reactions for limiting our metabolic modeling and representing the microenvironmental conditions in the plant nodules. In general, these can be classified as one of two categories. Class I includes those metabolites that can be exchanged between the bacteroid membrane and the plant environment. Among them, we included carbon dioxide (CO_2_), water (H_2_O), oxygen (O_2)_, malate (mal-L) and glutamate (glu-L). In addition, exchange reactions for nitrogen (n2), alanine (ala-L), aspartate (asp-L), succinate and ammonium (NH_4) _were included in the reconstruction for representing their possible bidirectional exchange from plant to bacteroids. On the other hand, metabolites in class II include those that contribute to the defining of internal frontiers in the bacteroids. Importantly, these sink reactions were included as a representation of metabolites originating from metabolic processes currently absent in the metabolic reconstruction. Thus, phosphate (pi), myo-inositol (inost), L-histidinol phosphate (hisp), palmitoyl-CoA (pmtcoa), dodecanoyl-CoA (dodecoa), decanoyl-CoA (decoa), octanoyl-CoA (otcoa) and hydrogen (h) fall in this classification.

### Definition of consistency coefficient

To assess the agreement between *in silico *predictions and interpretations suggested by high-throughput data, we defined a consistency coefficient, *η^Genes^*, that quantifies the fraction of genes that were predicted upregulated *in silico *and simultaneously detected or induced by proteome or transcriptome technologies. Simultaneously, we defined a consistency coefficient that quantifies the fraction of proteins enzymatically active that were predicted by constraint-base modeling and confirmed by high-throughput technology, *η^Enzymes^*. To proceed with this evaluation, we denoted ***E^j^_kegg_*(*G ^j^_kegg_*) **as the set of enzymes (genes) that form the *j-th *metabolic pathways in *KEGG *database, with *j-th *ranging from 1 to 22. Similarly, the set of enzymes (genes) that integrates the *i-th *metabolic pathway in the reconstruction and the set of enzymes detected by high-throughput data are denoted by ***E ^j^_Rec_*(*G ^j^_Rec_*) **and ***E ^j^_HT_*(*G ^j^_HT_*)**, respectively. Finally, the sets of enzymes and genes obtained from constraint-based modeling were denoted by ***E ^j^_iModel _***and ***G ^j^_iModel_***. More specifically, ***E ^j^_iModel _***and ***G ^j^_iModel _***sets were defined as those enzymes and genes participating in the active fluxes obtained from flux balance analysis. In order to evaluate and create a proper framework for comparison between *in silico *predictions and high-throughput data, we defined the consistency coefficient as the fraction of enzymes (genes) that were actively predicted *in silico *and were identified by high-throughput technology. This can be summed up in the following equations:

Both ratios range from zero to one and constitute our central parameter to assess and quantify the degree of coherence between constraint-based modeling and experimental data.

### *In silico *gene deletion analysis

Computational gene deletion analysis was used to quantify the effects that gene silencing has in supporting bacterial nitrogen fixation. Thus, once the gene to be switched off was selected, we identified its gene-protein reaction association and selected as zero its upper and lower bound in flux activity. Having made this adjustment, we applied flux balance analysis and obtained the new resulting objective function. In order to quantify the participation of this metabolic reaction in bacterial nitrogen fixation, we calculated the percentage of reduced activity of the mutated strain in comparison to the wild type, see Figure 3.

## Authors' contributions

OR-A conceived the metabolic reconstruction for *R. etli *and designed the computational analysis to simulate nitrogen fixation. ES realized microarray experiments, and OR-A contributed to their statistical analysis. ME and GM-B prepared the samples and accomplished the identification and processing in spectrometry analysis of all proteome data. YM guided the experimental cultivation for nodule preparation in *R etli*. SE conceived the study, design and analyzed the experimental data obtained from proteome and microarray technologies. All authors read and approved the final manuscript.

## Supplementary Material

Additional file 1 **Microarray Data Analysis**. This table shows those genes that were over expressed during bacteroid activity in nitrogen fixation. Click here for file

Additional file 2 **Proteome Data**. By using mass spectrometry, we identified a set of proteins during bacterial nitrogen fixation for *R. etli*. In each row, we named the protein and presented some of the parameter utilized for concluding the protein identify. Click here for file

Additional file 3 **Intersect between proteome and transcriptome**. Genes that were simultaneously identified by proteome and transcriptome technologies. Click here for file

Additional file 4 **This file contains an extended descriptive analysis deduced from the genes identified by proteome and transcriptome data**. Click here for file

Additional file 5 **(A) MA plot and representation of Metabolic activity**. In this figure we show the MA-plot obtained from microarray data and a selected representation of the metabolic activity predicted by FBA in some metabolic pathways: (B) TCA cycle, (C) purine and pyrimidine metabolism. Click here for file

Additional file 6 **Abbreviations**. This file enlists the main abbreviations used along the paper. Click here for file

Additional file 7 **Metabolic Reconstruction for *Rhizobium etli***. This table depicts the gene-protein-reaction association for *Rhizobium etli *metabolic reconstruction, *iOR450*. Overall the reconstruction contains 450 genes codifying for a set of enzymes participating in 405 metabolic reactions and 377 metabolites. Click here for file
