# Supplementary material for: Systems biology of bacterial nitrogen fixation: High-throughput technology and its integrative description with constraint-based modeling
Source: BMC Syst Biol. 2011 Jul 29;5:120. doi: 10.1186/1752-0509-5-120 (PMC3164627; doi:10.1186/1752-0509-5-120)
Supplement: Additional file 4 — This file contains an extended descriptive analysis deduced from the genes identified by proteome and transcriptome data. [file 1752-0509-5-120-S4.DOC]

**SUPPORTING INFORMATION.**

**Systems Biology of Bacterial Nitrogen Fixation: High throughput technology and its integrative description with constraint-based modeling.**

Osbaldo Resendis-Antonio^§^, Magdalena Hernández, Emmanuel Salazar, Sandra Contreras, Gabriel Martínez Batallar, Yolanda Mora and Sergio Encarnación^§^.

Programa de Genomica Funcional de Procariotes. Centro de Ciencias Genómicas-UNAM. Av. Universidad s/n, Col. Chamilpa, Cuernavaca Morelos, C.P. 62210. Mexico.

^§^Corresponding authors: encarnac@ccg.unam.mx, resendis@ccg.unam.mx.

**COMPLEMENTARY ANALYSIS OF HIGH THROUGHPUT DATA.**

To explore the metabolic activity underlying nitrogen fixation, we have accomplished an integrative description on *R.etli* bacteroids using transcriptome and proteome technology. As explained in the main text, this data set was essential to give a descriptive analysis of metabolism, extend the previous version of metabolic reconstruction in *R.etli* and assess its theoretical implications.

Microarray analysis for *R. etli* bacteroids led us to conclude that 689 genes, representing approximately 11% of its genome, were up-regulated in bacteroids respect to their expression during aerobic cell growth in minimal conditions, free-living condition. These results are consistent with previous microarray studies accomplished in *B. japonicum,* *S. meliloti,* *M. loti and R. leguminosarum* biovar viciae bacteroids[1-4]. Consistently with the non-growing physiological state prevailing in bacteroids, we observed that genes participating in translation machinery and some codifying enzymes for central metabolism down-regulate their expression respect to the free-living condition. Thus, 49 of the 55 ribosomal protein-encoding genes, genes required for translation initiation, elongation, termination and genes involved in the synthesis of amino-acyl tRNAs (*gltX*, *gatBAC*, *alaS*, *aspS*, and *proS*), were significatively repressed in bacteroids during nitrogen fixation. Despite their reduced activity, expression of these genes was not completely absent. Consequently, we were able to detect in bacteroids by proteome technology some elongation factors as Efp, TufB, FusA2 and PrfB.Likewise, *hisS*, *ileS*, g*lyS*, *leuS*, *argS, serS*, *gatB,* and *alaS2* which encoding hystidyl-tRNA, isoleucyl-tRNA, glycyl-tRNA, leucyl-tRNA, arginyl-tRNA, seryl-tRNA, glutamyl-tRNA and alanil-tRNA respectively, were repressed at the RNA level, but identified their protein products by proteomic analysis. In this contextual scheme, apparently *R. etli* bacteroids have sufficient metabolic energy and amino acids to support active transcription and protein synthesis. In agreement with these observations, amino acid tRNA synthetase, elongation factors, ribosomal proteins and transcription termination factor, constituted an important fraction of the identified proteins.

Overall, a total of 695 transcripts and 430 proteins (codifying by 293 unique IDs) were obtained for *R. etli* CFN42 bacteroids isolated from *P. vulgaris* nodules. In addition to the discussion exposed in the main text, additional physiological interpretations are highlighted here:

**TCA CYCLE, PYRUVATE DEHYDROGENASE AND ASPARTATE AMINO TRANSFERASE.** The pyruvate dehydrogenase complex catalyzes the oxidative decarboxylation of pyruvate to produce acetyl-CoA, linking glycolysis to the Krebs cycle. In *R. etli* and *S. meliloti*, the gene clusters encoding subunits of the pyruvate dehydrogenase (PDH) complex are very similar. The first three genes of the cluster, which probably constitute an operon [5] are: *pdhA1* (PDH (E1 component) α subunit), *pdhA2* (PDH (E1 component) β subunit) and *pdhB* (dihydrolipoamide acetyltransferase (E2 component).

By proteome technology, the PdhA2, PdhB and LpdACh2 (E3-component, dihydrolipoamide dehydrogenase proteins) were detected in *R.etli* nodule bacteria. This finding is consistent with the obtained for *S. meliloti* in which the *pdhA1* and *pdhA2* genes are highly expressed in bacteroid [6]. Pyruvate dehydrogenase (PDH) activity has been detected in *S. meliloti* nodule bacteria, and disruption of the aryl esterase gene impairs PDH complex activity and nitrogen fixation [7]. This suggests that the production of acetyl-CoA from malate using malic enzyme and pyruvate dehydrogenase, is important for funneling the carbon flux into the TCA cycle in the bacteroids [8]. However, in addition to the pyruvate dehydrogenase reaction, acetyl-CoA can be generated in bacteroids by different pathways which include, acetyl CoA synthetase, acetate kinase, acetocetyl CoA thiolases and acetaldehyde dehydrogenases. Of these, the last two types of proteins were detected by proteome technology, see additional file 3.

It has now been universally accepted that C_4_-dicarboxylates, particularly malate and succinate [9] are the principal carbon source during the nitrogen fixation for *R. etli* and *S. meliloti* bacteroids [10]*.* Proteome studies were no able to detected dicarboxylic transporters, however up-regulation of at least two putative dicarboxylic transporters were identified in nodule bacteria through microarray technology (*ypc00115* and *ypf00025*), see additional file 2. If C_4_-dicarboxylic acids are the only carbon source for bacteroids and are metabolized entirely by the TCA cycle, it would be expected that the TCA cycle must be completely operating.

On the other hand, we detected aconite hidratase (AcnA) through proteome technology. As we specified in the main text, aconitase mutants in *B. japonicum* still fixes nitrogen normally when inoculated onto soybeans, but this *acnA* mutants retain 30 % of the wild-type level of aconitase activity [11] suggesting the existence of a compensatory mechanism in nodules. Similarly, in *R. etli* CE3 isocitrate dehydrogenase and succinate dehydrogenase were not experimentally detected, however its presence have been confirmed in the crude extracts of *B. japonicum* bacteroids.

The consensus remains that, in nodules, the plant cytosol is the principal site for assimilation of ammonium into amino acids. Lodwig and associates [12] suggested that an amino acid cycle must operate where an amino acid such as glutamate, or a derivative of it, is supplied by the plant to the bacteroids through Aap (AapJ) and Bra (BraC2) amino acid transporters, both permeases were identified in *R. etli* bacteroids by proteome and *braC1* by transcriptomics. According with this proposal the bacteroid uses the amino acid to transaminate oxaloacetate or pyruvate to produce aspartate or alanine, respectively, and either or both of these amino acids are secreted. Consistent with this feedback, an important link between carbon and nitrogen metabolism is catalyzed by aspartate aminotransferase A (AatA). which catalyzes the reversible conversion of aspartate and 2-oxoglutarate to glutamate and oxaloacetate. In *R.etli,* two different aspartate aminotransferase were detected; AatA (ID RHE_CH02998) with proteomics and *aatCch* (ID RHE_CH01877) through transcriptomics. In addition, *S. meliloti* mutants in *aatA* (but not *aatB*) are unable to fix nitrogen suggesting a specific role for AatA in nodule bacteria [13].

**ENERGY TRANSFER.** An efficient production of energy is not only required for the normal metabolic function of the bacteroid, but also for maintaining the optimal rate of nitrogen fixation. Consistent with this function, the expression of electron transfer flavoproteins (ETF) were detected under nitrogen fixation: FixA (detected by proteomics and transcriptomics), FixB and FixX (confirmed by transcriptomics) and EtfAch (detected by proteomics). The *fixABCX* was the first characterized set of genes in the *Rhizobium* group, which are required for nitrogen fixation, in addition we observed expressed two ATP synthetases, α and β chains (AtpA and AtpD respectively), which were identified in the bacteroids by proteomics and *cpxA* was detected by transcriptomics. Also we detected through proteomics 3 electrophoretic protein identities from CpxP2 (cytochrome p450 monooxygenase protein) and the message detected induced by transcriptomics. This enzyme was described as essential in the oxidative, peroxidative, and reductive metabolism of numerous endogenous compounds (such as steroids, fatty acids, phytoalexins, and plant hormones) as well as xenobiotics in the environment, inclusive, in *Pseudomonas putida* was suggested to metabolize toxic compounds [14]. However in *B. japonicum* the mutant produced effective nodules on soybeans, even though the bacteroids contained no detectable P-450. This could imply that the cytochromes P-450 are not involved in an essential symbiotic function [15]. The precise role of P450 is ambiguous, we proposed that it functions as an alternate electron acceptor in the bacteroid and this does not exclude a putative role in mono-oxygenase and oxidoreduction reactions, possibly modifying the biological activity of its substrate and in this way detoxifying toxic compounds.

**CELLULAR PROTECTION.** The bacteria within the nodule is exposed to oxidants due the high rate of respiration required to provide energy for nitrogen fixation and for the autoxidation of leghaemoglobin, which generate high levels of active oxygen species in the nodule [16]. To overcome the harmful effect of reactive oxygen species (ROSs) in *R. etli* nodule-bacteria, we detected induced proteins involved in the detoxification of reactive oxygen species (ROSs) such as chromosomally-localized superoxide dismutase SodB (RHE_CH01203), the disruption of the *sodA* gene previously was shown to affect symbiotic efficiency of in alfalfa [17]. In addition, *ypch00606*, a probable anti-oxidant protein from AhpCTSA family and *ypch00400* a probable glutathione S-transferase protein were detected induced. Furthermore, two peroxiredoxins, RHE_CH00968) and RHE_PD00217 which is member of the NifA regulon [74] were also identified by proteomics. The peroxiredoxins function as antioxidants by reducing peroxides and alkyl hydroperoxides, thereby preventing the formation of hydroxyl radicals, which damage biomolecules. The role of peroxiredoxin in R. etli could therefore be protective for the bacteria within the nodule or, alternatively, could be used for modifying the plant response by influencing cell wall changes. On the other hand, catalase-peroxidase (*katG*), encoded on plasmid f was identified by proteomics. The *katG* mutant strain showed increase sensitivity to hydrogen peroxide in free-life, indicating an essential protective role in oxidative stress, however in symbiosis the mutant produced effective nodules and usual nitrogen fixation [18]. In this way, our results suggest the presence of two alternative defense mechanism against oxidative species. One of them prevailing in free-living conditions, with catalase-peroxidase as main component, while the other mechanisms in symbiosis is based on peroxiredoxins as participants, this latter similar to *S. meliloti* where was speculate that SodC might detoxify plant ROS, whereas the cytoplasmic SodA would be dedicated to the detoxification of ROS synthesized by the bacteria[19].

Two proteins (Hsp60)-type chaperonin, GroELch1 and GroELf were detected like multiple electrophoretic isoforms, *R. etli* have three chromosomal copies of *groEL* and a fourth is encoded on plasmid f. GroEL is one of the families of molecular chaperones that are involved in protein folding. In the nitrogen-fixing symbiont B. japonicum, five loci encode groEL. One of the genes is co-regulated with the nif and fix genes, implying some function within the nodule, although disruptions of any single B. japonicum groEL gene fail to impair nodulation or nitrogen fixation [20]. In the case of S. meliloti, cells containing a disruption of groELc are deficient in the activity of the nod gene transcriptional activators NodD1, NodD3 and SyrM, and the mutant cells elicit the formation of nodules that are Fix^−^ [21]. In the nodule, GroEL family members may have distinct roles, which could include assembly of nitrogenase, folding or assembly onto DNA of transcriptional regulators, stress response and translocation of proteins between the bacteroids and the plant.

**GLYCOLYSIS, GLUCONEOGENESIS AND PENTOSE PHOSPHATE PATHWAY.** Previous reports have shown that bacteroids lack an entire glycolytic cycle [22], however in *R.etli* bacteroids we detected 38 transcripts and 18 proteins whose functional classification fall in putative sugar transporters. In addition, our transcriptome analysis identified genes encoding several enzymes of glycolysis pathway, see additional file 2 and 3, an unexpected result given that the main carbon source in this compartment is dicarboxylic acids [10]. Combined analysis of proteomic and transcriptomics technologies led us to detect at least seven enzymes of the glycolytic pathway in the present study (additional file 2 and 3), in particular fructose bisphosphate aldolase (*fbaB*) was detected in nodule bacteria through both technologies.

In addition using proteomics methodology we detected two triosephosphate isomerases, TpiAch and TpiAf, glyceraldehyde 3-phosphate dehydrogenase (Gap), 2- phosphoglycerate dehydratase (enolase) and pyruvate kinase II (PykA). *Eno* mutants of *S. meliloti* fail to grow on TCA cycle intermediates or pyruvate [23]. Additionally, the transcript of one of several genes designated in the *R. etli* genome as phosphoglycerate mutase (in this case *pgm*,) was also detected by transcriptomics.

A notable metabolic feature in nodule bacteria was the activity of the protein PEP carboxykinase (*pckA*), a key gluconeogenic enzyme which was detected in multiple isoforms, see additional file 3. This enzyme catalyzes the first step in the conversion of tricarboxylic acid cycle intermediates to hexose sugars (gluconeogenesis), leading to the synthesis of glycogen. The presence and absence of this enzyme has been shown to have variable effects in nitrogen fixation activity and differentiation of the bacteria. For instance, in *Rhizobium sp.* strain NGR234 [24] its activity is required for growth on dicarboxylic acids. In *S. meliloti*, *pckA* expression is highly induced in minimal medium with succinate or arabinose as sole carbon source and is almost absent with glucose, sucrose or glycerol [1, 23, 24]. In *Rhizobium* NGR234, the *pckA* mutant strain has symbiotic phenotype host-plant dependent [24]. In bean plants, *R. etli* CE3 *pckA* mutants induce few nodules into which the infection threads do not appear to penetrate [25] .

In *R.etli* bacteroids the *NAD-malic* enzymes (Dme and Tme), which produces pyruvate directly from malate, were detected by proteomics. Interestingly, one of these enzymes (NAD^+^-malic enzyme) has been proven to be essential for nitrogen fixation in S. meliloti [26]. In addition, the 6-phosphogluconolactonase (Pgl), glucose 6-phosphate dehydrogenase (Zwf1), its chromosomal homolog, designated Zwf2, and one transaldolase (Tal) proteins of the pentose phosphate pathway, were detected by proteomics. In fast-growing rhizobia, the pentose phosphate pathway in combination with the Entner-Doudoroff pathway, are probably the major routes used for the metabolism of sugars [27]. These results open the possibility that, in addition to dicarboxylic acids, other carbon sources can participate during bacterial nitrogen fixation.

**ADDITIONAL METABOLIC CARBON PATHWAYS.** Poly-β-hydroxybutyrate (PHB) granules are produce by R. etli [28] and at least three components of the PHB pathway were detected in this study: 1) *PhbC* (poly beta-hydroxybutyrate polymerase protein), 2) one probable polyhydroxybutyrate depolymerase protein (*ypch00335*) detected by transcriptomics, and 3) the acetyl-CoA acetyltransferase (beta-ketothiolase, *PhbAch*) protein, see additional file 2. From a biochemical perspective, the production of PHB and fixation of nitrogen in bacteroids compete for the same energy and reductant sources, and therefore PHB synthesis in bacteroids must compete with nitrogen-fixation for photosynthate [28]. Also our results suggest the presence of glycogen as a storage compound in bacteroids, in this way we detected the *glgXch* and *glgXe* genes that encoded two glycosyl hydrolase (glycogen debranching) protein. Little is known about the role that glycogen could be playing during nodulation of legumes, however, it has been reported that in *R. tropici*, glycogen synthase (*glgA*) mutants have increased respiratory capacities and enhanced symbiotic performance [29]. Glycogen as PHB synthesis in free-living cells, are accumulated under growth-limiting conditions such as nitrogen-limitation [30], suggesting that glycogen metabolism may fulfill a similar role as the performed because of PHB metabolism, competing with nitrogenase for reductant, but the exact role of glycogen accumulation or degradation remains to be elucidated.

**REGULATORY PROTEINS.** According to the notion that two dimensional-electrophoresis (2-DE) is unable to detect low-abundance regulatory proteins; only three proteins were identified as transcriptional regulators. The regulatory proteins involved in nitrogen regulation were NtrX (two-component response regulator), PhoU (phosphate uptake transcriptional regulator) and Ypch01147 (probable transcriptional regulator protein, LysR family). On the other hand, FixL (two-component sensor histidine kinase protein) and two proteins classified as putative two-component histidine kinase proteins (Ypch00244 and Ypch00805), was also identified. Complementary, transcriptome analysis led us to detect at least 35 different probable and transcriptional regulator proteins, including *nodD2* (nod transcriptional regulator protein), seven nitrogen two components response regulator proteins, and 10 probable two component sensor histidine kinase. In this last group was included *ypd00005*, which was formerly identified as two-component sensor histidine kinase/response regulator and *ntrY* detected induced 4.83-fold. This gene was previously reported as the sensor element of the bacterial *ntrY/ntrX* two-component regulatory system involved in regulation of nitrogen metabolism, the other element of this regulatory system*,* NtrX, was detected by proteomics (see above). In *R. tropici,* the *ntrY* mutant strain was impaired in nodulation [31].

**PROTEINS INVOLVED IN TRANSPORT PROCESSES AND CELL SURFACE STRUCTURES.** *R.etli* encodes 713 ABC-transport system genes [5] and in this study we identified 95 transcripts and 52 proteins as ABC-ATP binding transporters. This set represents the 20.8 % of the ABC-transport systems detected in *R. etli*. The great variants of up-regulated ABC-transporters provide with some essential requirements for bacterial during the nitrogen fixation. We detected in *R. etli* bacteroids a large number of ABC-transporters involve in sugars transport; however, apparently sugars do not play a critical role as carbon source during the nitrogen fixation in symbiosis with legume plants. The components of ABC-transporters detected by transcriptomics and/or proteomics included fructose ABC transporter substrate binding protein encoded by *frcB* and fructose ABC transporter ATP-binding protein encoded by *frcA*. Both genes are part of *frcBCA* operon in *R. etli*. The same gene arrangement occurs in *S. meliloti*, where it was found that the *frc* system was targeted primarily for fructose uptake but also allowed the uptake of mannose and ribose. Mutants in this system were symbiotically proficient and an immunoblotting to detect the FrcB protein showed a very low level of expression in mature alfalfa nodule bacteroids [32]. Two *rbsAch1* and *rbsBch2* (ribose ABC transporter substrate binding protein) were detected, also the gene encoding sorbitol/manitol ABC transporter ATP-binding protein (*smoK)* was induced for *R. etli*, a similar induction rate as was reported for *S. meliloti* bacteroids [1]. In addition, we found that a probable ribose ABC transporter permease protein *ypf00013*, located on plasmid F, was up-regulated (151.69-fold). This gene is grouped with other genes, as *tpiAf* (triosephosphate isomerase), *rpiB* (ribose 5-phosphate isomerase) and probable ribose ABC-transporters.

One of the four genes contained in the thuEFGK cluster, which encoded a trehalose-maltose ABC transporter was up-regulated (8.4 fold) and its protein ThuE was too detected by proteomics. In *S. meliloti* a mutant in this gene was impaired in their ability to grow on either trehalose or maltose, but grew like wild-type on glucose or sucrose. A *thuE*-*lacZ* fusion showed that *thuE* was induced only by trehalose and not by cellobiose, glucose, maltopentose, maltose, mannitol or sucrose. ThuE mutants formed normal nitrogen-fixing nodules but were impaired for nodule formation when competed against the wild-type [33]. Similar phenotype was observed in *S. meliloti* mutants in components of alpha-glucosidase ABC transporter, which is encoded by *aglEFGAK* operon. In *S. meliloti*, this ABC-transport system was described to transport sucrose, maltose and trehalose and is induced primarily by sucrose and to a lesser degree by trehalose. In this study we detected by proteomics two members of this operon: AglE (substrate binding protein) and AglK (alpha-glucoside ABC transporter, ATP-binding protein) [33].

**Amino acids.** Of the elements identified as ABC-transporter proteins found in nodule bacteria, 13 were involved in amino acid transport, strongly suggesting that amino acid uptake is of particular importance to nodule bacteria. Different sets of amino acid ABC-transporters were detected during the nitrogen fixation, the general amino acid ABC-transporter protein AapJ (substrate binding protein) was detected by proteomics. The *aapJ* gene is part of *aapJQMP* operon which exists in many *Rhizobeaceas* and has been described in detail for *R. leguminosarum* [34, 35]. *BraC1* (transcriptomics) and *BraC2* (proteomics) members of the branched-chain amino acid ABC transporter were detected induced. In *R. etli* *braC1* is not clustered with other *bra* genes, while *braC2* is grouped with *braDEFG*, additionally we also detected by transcriptomics to *braF*. A similar arrangement occurs in *R. leguminosarum* where *braDEFG* has been shown to be necessary for alanine, histadine, leucine and arginine uptake, while *braC* mutants are effective for alanine uptake (but are deficient in the uptake of the other 3 amino acids) [36]. In *R. etli* *braC* mutants have not been studied, but *braD* and *braH* mutants were found deficient in glutamine uptake and respiration but proficient in nodulation and nitrogen fixation [25]. Additionally we were able to detect by transcriptomics and proteomics one probable amino acid ABC transporter, substrate-binding protein (Ypc0006) and eight probable amino acid ABC transporter permease proteins (one of them by proteomics and seven identified by transcriptomics).

**SMALL MOLECULES TRANSPORT. Metal ions.** Optimal nodule function requires a proper balanced of metabolites exchanged between the two symbiotic partners. This list of metabolites includes small molecules like nutrient ions as phosphate, iron and nikel. Phosphorus is an essential element for many biomolecules and plays an important role in many biological processes. Consistently with this fact, phosphate ABC-transporter, substrate-binding protein (PstS); phosphate ABC transporter, ATP-binding protein (PstB) and phosphate uptake transcriptional regulator (PhoU) were detected by proteomics. Furthermore, we identified up-regulated the peptide/nickel ABC-type transporter component OppA (substrate binding protein) and the AfuA3 iron (III) (ABC transporter, substrate-binding protein), both of them detected by proteomics and transcriptomics. Similarly, *ypf00126* (probable iron ABC transporter, substrate-binding protein), *ypf00127* (probable iron ABC transporter, permease protein), *fhuD* (ferrichrome-iron ABC transport, solute-binding protein), and *ypch00116* (putative iron-sulfur cluster binding protein), were strongly up-regulated in bacteroids. *SufC,* detected by proteomics, is an ATPase component of the SUF machinery, which is involved in the biosynthesis of Fe-S clusters [37]. The iron–sulfur (Fe–S) proteins are found in a variety of organisms and are required for a number of processes essential to cells, such as respiratory and regulation of gene expression. [[1]](http://www.sciencedirect.com/science?_ob=ArticleURL&_udi=B6T36-4HRDXBR-G&_user=945819&_coverDate=01%2F09%2F2006&_rdoc=1&_fmt=high&_orig=search&_sort=d&_docanchor=&view=c&_acct=C000048981&_version=1&_urlVersion=0&_userid=945819&md5=10b9529ddba2b307f644d17e1746cbb3#bbib1) H. Beinert, R.H. Holm and E. Münck, Iron–sulfur clusters: nature’s modular, multipurpose structures, *Science* **277** (1997), pp. 653–659. [**Full Text** via CrossRef](http://www.sciencedirect.com/science?_ob=RedirectURL&_method=outwardLink&_partnerName=3&_originPage=article&_zone=art_page&_targetURL=http%3A%2F%2Fdx.doi.org%2F10.1126%252Fscience.277.5326.653&_acct=C000048981&_version=1&_userid=945819&md5=986ae56c5dca9fc32c940a96cefa42bd) | [View Record in Scopus](http://www.sciencedirect.com/science?_ob=RedirectURL&_method=outwardLink&_partnerName=655&_originPage=article&_zone=art_page&_targetURL=http%3A%2F%2Fwww.scopus.com%2Finward%2Frecord.url%3Feid%3D2-s2.0-0030868605%26partnerID%3D10%26rel%3DR3.0.0%26md5%3D739ee9a78a1fa9ee07a65e4f49b9f9ab&_acct=C000048981&_version=1&_userid=945819&md5=0615945caac9557588adc7e7708753d7) | [Cited By in Scopus (624)](http://www.sciencedirect.com/science?_ob=RedirectURL&_method=outwardLink&_partnerName=656&_originPage=article&_zone=art_page&_targetURL=http%3A%2F%2Fwww.scopus.com%2Finward%2Fcitedby.url%3Feid%3D2-s2.0-0030868605%26partnerID%3D10%26rel%3DR3.0.0%26md5%3D739ee9a78a1fa9ee07a65e4f49b9f9ab&_acct=C000048981&_version=1&_userid=945819&md5=c596fee47f2a1b5bac678cc7bcd5091e)In plants, free iron is scarce and bacteria should acquire it from their host as occurs in a variety of pathogenic bacteria.

**Phosphonate**. Phosphonates are a class of organic phosphorus compounds characterized by a stable carbon–phosphorus (C–P) bond and their utilization by microbes provides a potential source of phosphorus for their growth. In bacteroids we detected a metabolic phosphonate protein (PhnM1) and the genes *ypch00055* and *ypch00056*, these latter identified as probable phosphonate ABC-transporter, substrate-binding proteins. Previous reports suggested than S. meliloti is able to growth with phosphonates as sole phosphorus sources [38]. On the other hand, *modA* which encode a putative molybdate transporter, was detected overexpress in bacteroids, it seems credible that bacteroids induce molybdate uptake systems to meet the molybdenum demand for nitrogenase biosynthesis. The importance of effective molybdate uptake systems for symbiotic nitrogen fixation has already been pointed out previously [39].

**Spermadine/Putrescine.** In *R. etli*, *potFGHI* occurs in an apparent operon and encode for a spermadine/putrescine ABC-transporter. In our study, transcript for *potH* and *potI*, genes encode permease components I and II, were detected induced. Furthermore, two spermidine/putrescine ABC transporters, the substrate-binding proteins *PotF* and *PotD* were also detected by proteomics. Spermadine and putrescine are polyamines which are known to be involved in the biosynthesis of nucleic acids and proteins, as well as to mediate cell growth and proliferation [40].

**Porins and Protein Export**. We detected different components for protein secretion among them, the protein export translocase component SecA *(*detected by proteomics) and *secE* by transcriptome analysis. The widespread *Sec* system enables the translocation of proteins across the inner membrane. In *Streptococcus gordonii* the *SecA* protein is not essential for cell viability but contribute to virulence [41]. In 8 addition, the canonical *Sec* system, SecE and SecG, in conjunction with SecY, form a heterotrimeric complex (*SecYEG*) that constitutes a pathway for polypeptide movement [42]. Also, TolB (protein transporter protein) belonging to the Tol-system was detected by proteomics, the Tol-system, of most gram-negative bacteria comprises five proteins, TolQ, TolR, TolA, TolB, and Pal. In E. coli, mutations in any of the tol-pal genes result in hypersensitivity to deleterious agents [43]. Additionally, we detected by transcriptomics two probable secretion proteins of the *HlyD* family (*ypd00053* and *ypch00443*). In addition, the *ropB2* gene encoding a outer membrane protein was detected induced in *R etli* bacteroids*,* in *R. leguminosarum* RopB mutants were reported severely affected during bacteroid formation [44].

**AMINO ACID METABOLISM.** Some amino acids are synthesized by *rhizobiaceas*, whereas others can be supplied by the host plant, whose details seems to be plant-type specific [45]. For instance, it has been reported that alfalfa provides arginine, cysteine, isoleucine, valine and tryptophan while cowpea and soybean provide histidin*e* [45]. In our high throughput study we can highlight the follows proteins and gens participating in amino acid metabolism:

**Arginine**. Arginine synthesis is essential for the nodulation of *P. vulgaris* by *R. etli* and its biosynthesis is required for the production of nodulation factors [46]. In *S. meliloti* Rmd201 mutants in *argB* and *argD* (ornithine auxotrophs), induced ineffective nodules whereas mutants in *argG* induced fully effective nodules in alfalfa plants [47]. Consistently, some genes and proteins participating in argininene pathway were identified: Acetylglutamate kinase (ArgB), acetylornithine aminotransferase (ArgD1), glutamate N-acetyltransferase/amino-acid N-acetyltransferase (ArgJ) and argininosuccinate synthase protein (ArgG2) were detected by proteomics, whereas *argG1* (argininosuccinate synthase protein) was identified by transcriptome analysis.

**Lysine.** In rhizobia, the lysine biosynthetic pathway has not yet been genetically characterized and, in our study some genes, *aatCch* and *dapB2* encoding aspartate-tyrosine-aromatic aminotransferase protein and dihydrodipicolinate reductase protein were induced. In bacteria, the diaminopimelate (*DAP*) and lysine biosynthesis pathways share one key enzyme, the dihydrodipicolinate reductase (*DapB*). In *S. meliloti* strain GR4, *dapB* mutant strains nodulate alfalfa in the same way as the wild type strain, but this mutant is not lysine auxotroph. This finding suggest the presence of additional *dap* genes in strains like GR4 (as happens in *R. etli* CE3) representing an alternative pathway for the synthesis of DAP and lysine. Additionally, aspartate-ß-semialdehyde dehydrogenase (*Asd)*, which is the second enzyme in the lysine/homoserine biosynthetic pathways and succcinyl-diaminopimelate desuccinylase (DapE1*)*, involved in lysine biosynthesis, were detected by proteomics.

**Tyrosine, tryptophan and phenylalanine.** Two genes involved in tyrosine, tryptophan and phenylalanine biosynthesis; *aroQ2* encoded dehydroquinate dehydratase and *aroA* (phosphoshikimate 1-carboxyvinyltransferase protein), were detected up-regulated in *R. etli* bacteroids. Also, 3- tryptophan synthase beta chain protein (TrpB), part of the tryptophan synthesis pathway was detected by proteomics. In *R. etli* a mutant strain in *trpB* gene (CTNUX4) was able to induce only small, slightly pink, ineffective (Fix¯) nodules. However, under free-living conditions, strain CTNUX4 was unable to produce flavonoid-inducible lipo-chitin oligosaccharides (Nod factors), unless tryptophan was added to the growth medium. These data and histological observations indicate that the lack of tryptophan biosynthesis affects the symbiotic behavior of *R. etli* [48].

**Cysteine**. Several enzymes have evolved as sensors in signal transduction pathways for participating in the control gene expression. This is the case of CysK (cysteine synthase), which was detected by proteomics *in R.etli* bacteroids. In bacteria, some archea and plants, cysteine biosynthesis proceeds via a two-step pathway involving cysteine synthase (CysK) and serine acetyl-transferase (CysE). CysE catalyzes the acetylation of l-serine by acetyl-CoA to give O-acetyl-l-serine (OAS). The OAS-thiollyase, CysK, then converts OAS and sulfide into l-cysteine and acetate. According to its catalytic mechanism, CysK belongs to the fold type II group of pyridoxal 5′-phosphate (PLP)-dependent enzymes. In Salmonella typhimurium, E. coli, Haemophilus influenzae, Mycobacterium tuberculosis, and higher plants, *CysE* and *CysK* form a bienzyme complex, called also cysteine synthase. Recently, Tanous *et al*.[49] identified the master regulator of cysteine metabolism in Bacillus subtilis, CymR, this group proposed a model, by which CysK positively regulates CymR in sensing the bacterial cysteine pool. In *R. etli* the precise role of this protein is still unknown. Similarly to CysK, PutA (proline dehydrogenase-aldehyde dehydrogenase) was detected induced in bacteroids. These bifunctional proteins, apparently active as well in metabolism as in regulation are called trigger enzymes [49]. The metabolic role of PutA described in *S. meliloti* is to catalyze the oxidation of proline to glutamate, a *putA* null mutation leads to a significant reduction of alfalfa root colonization [50].

**Methionine and homoserine**. Three elements of the homoserine and methionine biosynthesis were identified by high throughput technology: *mdeAe* (methionine gamma-lyase protein) was detected up-regulated by transcriptomics and two proteins, the aspartate-B-semialdehyde dehydrogenase protein (Asd) and homoserine dehydrogenase protein (ThrA) were detected in nodule bacterois. The symbiotic phenotype of Met^−^ auxotrophs is a strain dependent property [51, 52]. In *R. etli* the *metZ* mutant was unable to produce flavonoid-inducible *Nod* factors, and thereby unable to induce nodules on the roots of *P. vulgaris* [48]. An adenosylhomocysteinase (AhcY), was also detected by proteomics; this protein is involved in either selenohomocysteine production from selenomethionine, or in producing homocysteine from methionine. In bacteroid a dihydrolipoamide dehydrogenase (LpdAc), localized in plasmid C and participate in valine, leucine and isoleucine degradation was detected in this study. The *mccB* message*,* (methylcrotonyl-CoA carboxylase biotin-containing subunit), which also is part of this degradative pathway, was also detected induced.

Several genes participating in different amino acid biosynthetic pathways were detected in this study and mutants in some *Rhizobiaceas* suggest their essentiality for the establishment of symbiosis and functional nitrogen fixation.

**POLYSCCHARIDE SYNTHESIS AND REGULATION.** *Rhizobia* produce polysaccharides, such as exopolysaccharides (EPSs), capsular polysaccharides (KPSs), lipopolysaccharides (LPSs), and cyclic β-(1,2)-glucans, all of them necessary for establishing symbiotic associations [53]. In *R. etli* bacteroid, we were able to detected two EPS biosynthesis type of genes (*exo* and *pss*), however, also a possible negative regulator of EPS synthesis (*exoR).*  ExoR was described previously as a negative regulator of succinoglycan synthesis [54], and more recently, exoR was shown to interact genetically with exoS/chvI and encode a periplasmic protein, suggesting that *ExoR* represents a new type of bacterial two-component system inhibitor, specifically over ExoS/ChvI. The functions regulated by ExoS/ChvI include exopolysaccharide production, biofilm formation, motility, and nutrient utilization [55]. On the other hand, we detected by transcriptomics the *pssV* gene (exopolysaccharide production protein), and also we identified in bacteroids the exopolysaccharide export protein PssN and detected up-regulated *pssO* (5.52-fold) which in *R. leguminosarum* bv*. trifoli* [56] and *R. etli* is part of the *pssTNOP* gene cluster. These genes are implicated in exopolysaccharide synthesis and transport [56]. *PssO* recently was described in *Rhizobium leguminosarum* bv. *trifolii* strain RtTA1 as a unique protein important for EPS production, hypothesizing functions as a periplasmic “chaperon” coating the EPS polymer and protecting it from glycanases, and/or being co-transported with the polysaccharide through a channel formed in the outer membrane [57]. Furthermore, the UTP-glucose-1-phosphate uridylyltransferase protein (ExoN), that participate in the synthesis of succinoglycans, was identified in this study. In *S. meliloti* an *exoN* mutant strain synthesize only 20% of the succinoglycan produced by the wild-type strain however is symbiotically proficient [58]. On the other hand, cellulose synthase encoded by *celA* was detected induced in symbiosis, in *R. etli*, this gene is clustered with those encoding a UDP-Glc-4-epimerase (*galE1*) and a cellulose H endoglycanase, in *R. leguminosarum* was previously shown that *celA* mutant attached and formed normal biofilms *in vitro*, but it did not form a biofilm on root hairs even when attachment occured. In this way, the cellulose-dependent biofilm on root hairs appears not to be critical for nodulation, because the *celA* mutant competed with the wild-type for nodule infection [59].

**OTHER CELLULAR FUNCTIONS.** A wide variety of *hem* related genes were detected up-regulated, including *ypd00056* (putative biosynthetic heme) and *hemNd2* (coproporphyrinogen III oxidase protein). *HemNd2* is involved in porphyrin biosynthesis catalyzing the oxidative decarboxylation of the propionate side chains of rings A and B of coproporphyrinogen III to form protoporphyrinogen IX. In addition, we observed induced the genes *cycX* (probable heme transporter protein) and *ctaB* (protoheme IX farnesyltransferase protein). Both genes were reported essentials for the formation of all cellular c-type cytochromes in *B. japonicum* and *Bordetella pertussis* respectively [60, 61]. Transcript for tyrosinase monophenol monooxygenase, encoded by *melA* on the *R. etli* pSym, and which participates in the conversion of tyrosine to melanin [62], was detected induced and also was identified by proteomics in multiple electrophoretic isoforms. The function melanin in *rhizobia* is unknown [63], although it could participate as electron donator/acceptor [64].

Two peptidyl prolyl cis-trans isomerases (PpiB and PpiD2*)* was identified by proteomics. Both proteins have a role in protein folding, however their up-regulation can have an importance or specific role in the nitrogen fixation process [65]**.** Transcript for *casA*, encoding calsymin, a calcium-binding protein. In *R. etli* CNPAF512, *casA* was expressed during colonizationinfection of *P. vulgaris*, and in nodules, *casA* mutants were significantly less symbiotically efficient than the wild-type [66]. Calcium has a role in a large number of cellular processes, and may be of particular importance in the establishment of symbiosis as calcium spiking is one of the earliest responses of plant root hairs to Nod factors [67].

**GENES OF UNKNOWN OR AMBIGUOUS FUNCTION.** The bacteroid protein S (*bacS*) transcript was detected up-regulated inbacteroids. *R. etli* contains three pSym-borne *bacS* genes, each of which apparently produces a protein product. They are not expressed in rich medium but are during symbiosis. Mutants disrupted or deleted in all three genes did not produce the BacS polypeptide but were Nod+ and Fix+ on *P. vulgaris* [68]. In *R. etli* genome, 13 adenylate cyclase and probable adenylate cyclase-encoding genes have been annotated [5], one of them was detected by proteomics (Ypch00842*).* In addition, *cyaFch* and two probable adenylate cyclases (*ypch00869* and *ype00080*) were detected up-regulated,. Cyclic nucleotides in prokaryotes, particularly cAMP, are mostly known as regulators of catabolic functions. In other cases they are involved in communication with host cells. However the role of these cyclases in *R.etli* remains to be elucidated [69].

**BIBLIOGRAPHY:**

1. Barnett MJ, Toman CJ, Fisher RF, Long SR: **A dual-genome Symbiosis Chip for coordinate study of signal exchange and development in a prokaryote-host interaction.** *Proc Natl Acad Sci U S A* 2004, **101:**16636-16641.

2. Becker A, Berges H, Krol E, Bruand C, Ruberg S, Capela D, Lauber E, Meilhoc E, Ampe F, de Bruijn FJ, *et al*: **Global changes in gene expression in *Sinorhizobium meliloti* 1021 under microoxic and symbiotic conditions.** *Mol Plant Microbe Interact* 2004, **17:**292-303.

3. Uchiumi T, Ohwada T, Itakura M, Mitsui H, Nukui N, Dawadi P, Kaneko T, Tabata S, Yokoyama T, Tejima K, *et al*: **Expression islands clustered on the symbiosis island of the *Mesorhizobium loti* genome.** *J Bacteriol* 2004, **186:**2439-2448.

4. Karunakaran R, Ramachandran VK, Seaman JC, East AK, Mouhsine B, Mauchline TH, Prell J, Skeffington A, Poole PS: **Transcriptomic analysis of *Rhizobium leguminosarum* biovar *viciae* in symbiosis with host plants *Pisum sativum* and *Vicia cracca*.** *J Bacteriol* 2009, **191:**4002-4014.

5. Gonzalez V, Santamaria RI, Bustos P, Hernandez-Gonzalez I, Medrano-Soto A, Moreno-Hagelsieb G, Janga SC, Ramirez MA, Jimenez-Jacinto V, Collado-Vides J, Davila G: **The partitioned *Rhizobium etli* genome: genetic and metabolic redundancy in seven interacting replicons.** *Proc Natl Acad Sci U S A* 2006, **103:**3834-3839.

6. Cabanes D, Boistard P, Batut J: **Symbiotic induction of pyruvate dehydrogenase genes from *Sinorhizobium meliloti.*** *Mol Plant Microbe Interact* 2000, **13:**483-493.

7. Soto MJ, Sanjuan J, Olivares J: **The disruption of a gene encoding a putative arylesterase impairs pyruvate dehydrogenase complex activity and nitrogen fixation in *Sinorhizobium meliloti*.** *Mol Plant Microbe Interact* 2001, **14:**811-815.

8. Poole P, Allaway D: **Carbon and nitrogen metabolism in Rhizobium.** *Adv Microb Physiol* 2000, **43:**117-163.

9. Watson RJ, Chan YK, Wheatcroft R, Yang AF, Han SH: ***Rhizobium meliloti* genes required for C4-dicarboxylate transport and symbiotic nitrogen fixation are located on a megaplasmid.** *J Bacteriol* 1988, **170:**927-934.

10. Djordjevic MA: ***Sinorhizobium meliloti* metabolism in the root nodule: a proteomic perspective.** *Proteomics* 2004, **4:**1859-1872.

11. Thony-Meyer L, Kunzler P: **The *Bradyrhizobium japonicum* aconitase gene (acnA) is important for free-living growth but not for an effective root nodule symbiosis.** *J Bacteriol* 1996, **178:**6166-6172.

12. Lodwig EM, Hosie AH, Bourdes A, Findlay K, Allaway D, Karunakaran R, Downie JA, Poole PS: **Amino-acid cycling drives nitrogen fixation in the legume-Rhizobium symbiosis.** *Nature* 2003, **422:**722-726.

13. Watson RJ, Rastogi VK: **Cloning and nucleotide sequencing of *Rhizobium meliloti* aminotransferase genes: an aspartate aminotransferase required for symbiotic nitrogen fixation is atypical.** *J Bacteriol* 1993, **175:**1919-1928.

14. Mueller EJ, Loida PJ, SG S: *Twenty-five years of P450cam research.* Plenum Press, New York. edn; 1995.

15. Tully RE, Keister DL: **Cloning and Mutagenesis of a Cytochrome P-450 Locus from *Bradyrhizobium japonicum* That Is Expressed Anaerobically and Symbiotically.** *Appl Environ Microbiol* 1993, **59:**4136-4142.

16. Dalton D: *Antioxidant defenses of plants and fungi. In Oxidative Stress and Antioxidant Defenses in Biology.*: New York: Chapman & Hall; 1995.

17. Santos R, Herouart D, Puppo A, Touati D: **Critical protective role of bacterial superoxide dismutase in rhizobium-legume symbiosis.** *Mol Microbiol* 2000, **38:**750-759.

18. Vargas Mdel C, Encarnacion S, Davalos A, Reyes-Perez A, Mora Y, Garcia-de los Santos A, Brom S, Mora J: **Only one catalase, *katG*, is detectable in *Rhizobium etli*, and is encoded along with the regulator OxyR on a plasmid replicon.** *Microbiology* 2003, **149:**1165-1176.

19. Ampe F, Kiss E, Sabourdy F, Batut J: **Transcriptome analysis of *Sinorhizobium meliloti* during symbiosis.** *Genome Biol* 2003, **4:**R15.

20. Fischer HM, Babst M, Kaspar T, Acuna G, Arigoni F, Hennecke H: **One member of a gro-ESL-like chaperonin multigene family in *Bradyrhizobium japonicum* is co-regulated with symbiotic nitrogen fixation genes.** *EMBO J* 1993, **12:**2901-2912.

21. Ogawa J, Long SR: **The *Rhizobium meliloti* groELc locus is required for regulation of early nod genes by the transcription activator NodD.** *Genes Dev* 1995, **9:**714-729.

22. Hudman JF, Glenn AR: **Glucose uptake by free living and bacteroid forms of *Rhizobium leguminosarum*.** *Archives of Microbiology* 1980, **128:**72-77.

23. Finan TM, Oresnik I, Bottacin A: **Mutants of *Rhizobium meliloti* defective in succinate metabolism.** *J Bacteriol* 1988, **170:**3396-3403.

24. Osteras M, Finan TM, Stanley J: **Site-directed mutagenesis and DNA sequence of *pckA* of *Rhizobium* NGR234, encoding phosphoenolpyruvate carboxykinase: gluconeogenesis and host-dependent symbiotic phenotype.** *Mol Gen Genet* 1991, **230:**257-269.

25. Tate R, Ferraioli S, Filosa S, Cermola M, Riccio A, Iaccarino M, Patriarca EJ: **Glutamine utilization by *Rhizobium etli*.** *Mol Plant Microbe Interact* 2004, **17:**720-728.

26. Driscoll BT, Finan TM: **NAD(+)-dependent malic enzyme of *Rhizobium meliloti* is required for symbiotic nitrogen fixation.** *Mol Microbiol* 1993, **7:**865-873.

27. Romanov VI, Hernandez-Lucas I, Martinez-Romero E: **Carbon Metabolism Enzymes of *Rhizobium tropici* Cultures and Bacteroids.** *Appl Environ Microbiol* 1994, **60:**2339-2342.

28. Cevallos MA, Encarnacion S, Leija A, Mora Y, Mora J: **Genetic and physiological characterization of a *Rhizobium etli* mutant strain unable to synthesize poly-beta-hydroxybutyrate.** *J Bacteriol* 1996, **178:**1646-1654.

29. Marroqui S, Zorreguieta A, Santamaria C, Temprano F, Soberon M, Megias M, Downie JA: **Enhanced symbiotic performance by *Rhizobium tropici* glycogen synthase mutants.** *J Bacteriol* 2001, **183:**854-864.

30. Zevenhuizen LP: **Cellular glycogen, beta-1,2,-glucan, poly beta-hydroxybutyric acid and extracellular polysaccharides in fast-growing species of Rhizobium.** *Antonie Van Leeuwenhoek* 1981, **47:**481-497.

31. Nogales J, Campos R, BenAbdelkhalek H, Olivares J, Lluch C, Sanjuan J: ***Rhizobium tropici* genes involved in free-living salt tolerance are required for the establishment of efficient nitrogen-fixing symbiosis with Phaseolus vulgaris.** *Mol Plant Microbe Interact* 2002, **15:**225-232.

32. Lambert A, Osteras M, Mandon K, Poggi MC, Le Rudulier D: **Fructose uptake in *Sinorhizobium meliloti* is mediated by a high-affinity ATP-binding cassette transport system.** *J Bacteriol* 2001, **183:**4709-4717.

33. Jensen JB, Peters NK, Bhuvaneswari TV: **Redundancy in periplasmic binding protein-dependent transport systems for trehalose, sucrose, and maltose in *Sinorhizobium meliloti.*** *J Bacteriol* 2002, **184:**2978-2986.

34. Walshaw DL, Poole PS: **The general L-amino acid permease of *Rhizobium leguminosarum* is an ABC uptake system that also influences efflux of solutes.** *Mol Microbiol* 1996, **21:**1239-1252.

35. Walshaw DL, Wilkinson A, Mundy M, Smith M, Poole PS: **Regulation of the TCA cycle and the general amino acid permease by overflow metabolism in *Rhizobium leguminosarum*.** *Microbiology* 1997, **143 ( Pt 7):**2209-2221.

36. Hosie AH, Allaway D, Galloway CS, Dunsby HA, Poole PS: ***Rhizobium leguminosarum* has a second general amino acid permease with unusually broad substrate specificity and high similarity to branched-chain amino acid transporters (Bra/LIV) of the ABC family.** *J Bacteriol* 2002, **184:**4071-4080.

37. Kitaoka S, Wada K, Hasegawa Y, Minami Y, Fukuyama K, Takahashi Y: **Crystal structure of Escherichia coli SufC, an ABC-type ATPase component of the SUF iron-sulfur cluster assembly machinery.** *FEBS Lett* 2006, **580:**137-143.

38. Parker GF, Higgins TP, Hawkes T, Robson RL: ***Rhizobium (Sinorhizobium) meliloti phn* genes: characterization and identification of their protein products.** *J Bacteriol* 1999, **181:**389-395.

39. Delgado MJ, Tresierra-Ayala A, Talbi C, Bedmar EJ: **Functional characterization of the *Bradyrhizobium japonicum modA* and *modB* genes involved in molybdenum transport.** *Microbiology* 2006, **152:**199-207.

40. Igarashi K, Kashiwagi K: **Polyamine transport in bacteria and yeast.** *Biochem J* 1999, **344 Pt 3:**633-642.

41. Braunstein M, Brown AM, Kurtz S, Jacobs WR, Jr.: **Two nonredundant SecA homologues function in mycobacteria.** *J Bacteriol* 2001, **183:**6979-6990.

42. Mori H, Ito K: **The Sec protein-translocation pathway.** *Trends Microbiol* 2001, **9:**494-500.

43. Lazzaroni JC, Germon P, Ray MC, Vianney A: **The Tol proteins of *Escherichia coli* and their involvement in the uptake of biomolecules and outer membrane stability.** *FEMS Microbiol Lett* 1999, **177:**191-197.

44. de Maagd R, de Rijk R, Mulders IH, Lugtenberg BJ: **Immunological characterization of *Rhizobium leguminosarum* outer membrane antigens by use of polyclonal and monoclonal antibodies.** *J Bacteriol* 1989, **171:**1136-1142.

45. Randhawa GS, Hassani R: **Role of rhizobial biosynthetic pathways of amino acids, nucleotide bases and vitamins in symbiosis.** *Indian J Exp Biol* 2002, **40:**755-764.

46. Ferraioli S, Tate R, Caputo E, Lamberti A, Riccio A, Patriarca EJ: ***The Rhizobium etli argC* gene is essential for Arginine biosynthesis and nodulation of Phaseolus vulgaris.** *Mol Plant Microbe Interact* 2001, **14:**250-254.

47. Kumar A, Vij N, Randhawa GS: **Isolation and symbiotic characterization of transposon Tn5-induced arginine auxotrophs of *Sinorhizobium meliloti*.** *Indian J Exp Biol* 2003, **41:**1198-1204.

48. Tate R, Riccio A, Caputo E, Iaccarino M, Patriarca EJ: **The *Rhizobium etli metZ* gene is essential for methionine biosynthesis and nodulation of *Phaseolus vulgaris*.** *Mol Plant Microbe Interact* 1999, **12:**24-34.

49. Tanous C, Soutourina O, Raynal B, Hullo MF, Mervelet P, Gilles AM, Noirot P, Danchin A, England P, Martin-Verstraete I: **The CymR regulator in complex with the enzyme CysK controls cysteine metabolism in *Bacillus subtilis*.** *J Biol Chem* 2008, **283:**35551-35560.

50. Soto MJ, Jimenez-Zurdo JI, van Dillewijn P, Toro N: ***Sinorhizobium meliloti putA* gene regulation: a new model within the family Rhizobiaceae.** *J Bacteriol* 2000, **182:**1935-1941.

51. Pain A: **Symbiotic properties of antibiotic-resistant and auxotrophic mutants of *Rhizobium leguminosarum*.** *J Appl Bacteriol* 1979, **47:**53-64.

52. Kerppola TK, Kahn ML: **Symbiotic phenotypes of auxotrophic mutants of *Rhizobium meliloti* 104A14.** *J Gen Microbiol* 1988, **134:**913-919.

53. Kannenberg EL, Brewin NJ: **Host-plant invasion by Rhizobium: the role of cell-surface components.** *Trends Microbiol* 1994, **2:**277-283.

54. Doherty D, Leigh JA, Glazebrook J, Walker GC: ***Rhizobium meliloti* mutants that overproduce the R. meliloti acidic calcofluor-binding exopolysaccharide.** *J Bacteriol* 1988, **170:**4249-4256.

55. Chen EJ, Sabio EA, Long SR: **The periplasmic regulator ExoR inhibits ExoS/ChvI two-component signalling in *Sinorhizobium meliloti.*** *Mol Microbiol* 2008, **69:**1290-1303.

56. Marczak M, Mazur A, Krol JE, Gruszecki WI, Skorupska A: **Lipoprotein PssN of *Rhizobium leguminosarum* bv. *trifolii*: subcellular localization and possible involvement in exopolysaccharide export.** *J Bacteriol* 2006, **188:**6943-6952.

57. Marczak M, Mazur A, Gruszecki WI, Skorupska A: **PssO, a unique extracellular protein important for exopolysaccharide synthesis in *Rhizobium leguminosarum* bv*. trifolii.*** *Biochimie* 2008, **90:**1781-1790.

58. Long S, Reed JW, Himawan J, Walker GC: **Genetic analysis of a cluster of genes required for synthesis of the calcofluor-binding exopolysaccharide of *Rhizobium meliloti*.** *J Bacteriol* 1988, **170:**4239-4248.

59. Williams A, Wilkinson A, Krehenbrink M, Russo DM, Zorreguieta A, Downie JA: **Glucomannan-mediated attachment of *Rhizobium leguminosarum* to pea root hairs is required for competitive nodule infection.** *J Bacteriol* 2008, **190:**4706-4715.

60. Ramseier TM, Winteler HV, Hennecke H: **Discovery and sequence analysis of bacterial genes involved in the biogenesis of c-type cytochromes.** *J Biol Chem* 1991, **266:**7793-7803.

61. Feissner RE, Beckett CS, Loughman JA, Kranz RG: **Mutations in cytochrome assembly and periplasmic redox pathways in *Bordetella pertussis*.** *J Bacteriol* 2005, **187:**3941-3949.

62. Coon SL, Kotob S, Jarvis BB, Wang S, Fuqua WC, Weiner RM: **Homogentisic acid is the product of MelA, which mediates melanogenesis in the marine bacterium *Shewanella colwelliana* D.** *Appl Environ Microbiol* 1994, **60:**3006-3010.

63. Mercado-Blanco J, Garcia F, Fernandez-Lopez M, Olivares J: **Melanin production by *Rhizobium meliloti* GR4 is linked to nonsymbiotic plasmid pRmeGR4b: cloning, sequencing, and expression of the tyrosinase gene *mepA*.** *J Bacteriol* 1993, **175:**5403-5410.

64. Turick CE, Tisa LS, Caccavo F, Jr.: **Melanin production and use as a soluble electron shuttle for Fe(III) oxide reduction and as a terminal electron acceptor by *Shewanella algae* BrY.** *Appl Environ Microbiol* 2002, **68:**2436-2444.

65. Rathbun KM, Hall JE, Thompson SA: **Cj0596 is a periplasmic peptidyl prolyl cis-trans isomerase involved in *Campylobacter jejuni* motility, invasion, and colonization.** *BMC Microbiol* 2009, **9:**160.

66. Xi C, Schoeters E, Vanderleyden J, Michiels J: **Symbiosis-specific expression of *Rhizobium etli casA* encoding a secreted calmodulin-related protein.** *Proc Natl Acad Sci U S A* 2000, **97:**11114-11119.

67. Ehrhardt DW, Wais R, Long SR: **Calcium spiking in plant root hairs responding to *Rhizobium nodulation* signals.** *Cell* 1996, **85:**673-681.

68. Jahn OJ, Davila G, Romero D, Noel KD: **BacS: an abundant bacteroid protein in *Rhizobium etli* whose expression ex planta requires *nifA*.** *Mol Plant Microbe Interact* 2003, **16:**65-73.

69. Tellez-Sosa J, Soberon N, Vega-Segura A, Torres-Marquez ME, Cevallos MA: **The *Rhizobium etli cyaC* product: characterization of a novel adenylate cyclase class.** *J Bacteriol* 2002, **184:**3560-3568.

70. Salazar E, Diaz-Mejia JJ, Moreno-Hagelsieb G, Martinez-Batallar G, Mora Y, Mora J, Encarnacion S: **Characterization of the NifA-RpoN regulon in *Rhizobium etli* in free life and in symbiosis with *Phaseolus vulgaris*.** *Appl Environ Microbiol*, **76:**4510-4520.
