# Supplementary figures and images for: Systems biology of bacterial nitrogen fixation: High-throughput technology and its integrative description with constraint-based modeling
Source: BMC Syst Biol. 2011 Jul 29;5:120. doi: 10.1186/1752-0509-5-120 (PMC3164627; doi:10.1186/1752-0509-5-120)

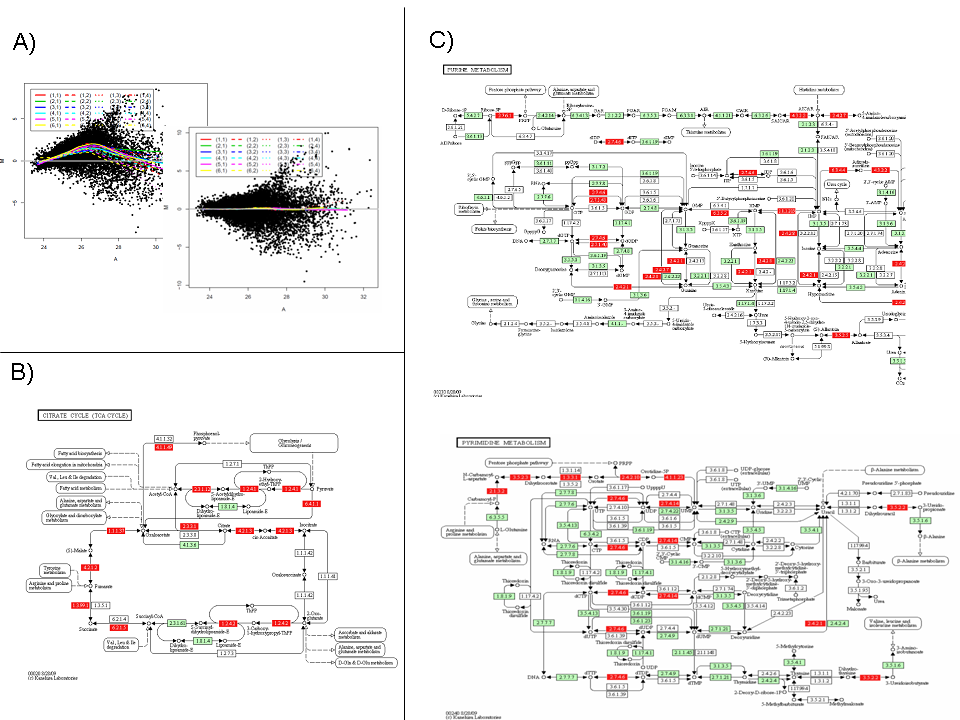

Supplement: Additional file 5 — (A) MA plot and representation of Metabolic activity. In this figure we show the MA-plot obtained from microarray data and a selected representation of the metabolic activity predicted by FBA in some metabolic pathways: (B) TCA cycle, (C) purine and pyrimidine metabolism. [file 1752-0509-5-120-S5.TIFF]
