# Supplementary material for: Systems biology of bacterial nitrogen fixation: High-throughput technology and its integrative description with constraint-based modeling
Source: BMC Syst Biol. 2011 Jul 29;5:120. doi: 10.1186/1752-0509-5-120 (PMC3164627; doi:10.1186/1752-0509-5-120)
Supplement: Additional file 6 — Abbreviations. This file enlists the main abbreviations used along the paper. [file 1752-0509-5-120-S6.DOC]

**ABBREVIATION PAGE.**

*AcnA aconitase hidratase.*

***E ^j^_kegg_*** Set of enzymes that integrate the *j-esime* metabolic pathways in *KEGG* database.

***E ^j^_Model_*** The set of enzymes that constraint-based modeling predict as active.

*FumC* Fumarate hydratase (fumarase A)

*FumB* Fumarate hydratase (fumarase B)

Gap *glyceraldehyde 3-phosphate dehydrogenase*

*Gnd* *6-phosphogluconate* *dehydrogenase.*

***G ^j^_kegg_***  The set of genes that integrate the *j-esime* metabolic pathways in *KEGG*.

***G ^j^_Model_***, The set of genes that constraint-based modeling predict as active.

*iOR*363 First *in silico* Metabolic Reconstruction of Rhizobium etli.

*iOR*450 Second *in silico* Metabolic Reconstruction of Rhizobium etli.

*Icd isocitrate dehydrogenase.*

*iscN* gene codifying for Fe-S cofactor nitrogenase synthesis protein.

*LpdAch* dihydrolipoamide dehydrogenase.

*Mdh* [malate dehydrogenase (oxaloacetate-decarboxylating) (NADP+)activity](http://amigo.geneontology.org/cgi-bin/amigo/go.cgi?view=details&depth=1&query=4473)*.*

*η* Coefficient of Consistency.

*η _Genes_* Coefficient of consistency for genes.

*η _Enzyme_* Coefficient of consistency for enzymes.

*PDH pyruvate dehydrogenase*

*pykA pyruvate kinase* II.

*pgm phosphoglycerate mutase aldolase.*

*pckA PEP* *carboxykinase.*

*pgl 6-phosphogluconolactonase.*

*PHB Poly-β-hydroxybutyrate.*

*R. etli Rhizobium etli.*

*S. meliloti Sinorhizobium meliloti.*

*SucB* dihydrolipoamide succinyltransferase (E2).

*SucA* subunit of alpha-ketoglutarate dehydrogenase.

*SucC* succinyl-CoA synthetase, beta subunit.

*Tal* *transaldolase*

*TpiA triosephosphate isomerase.*

*Zwf1 6-phosphate dehydrogenase.*

*FBA Flux Balanca Analysis*
